# Formation of characteristic aroma compounds in walnut kernels during thermal processing and their potential recognition by human olfactory receptors

**DOI:** 10.1016/j.fochx.2026.104142

**Published:** 2026-07-09

**Authors:** Yishen Cheng, Shanxing Gao, Lei Zhang, Chenyan Lv, Jiachen Zang

**Affiliations:** College of Food Science and Nutritional Engineering, China Agricultural University, Beijing 100083, China

**Keywords:** Thermal processing, Walnut aroma, Sensory attributes, Olfactory receptors, Molecular docking

## Abstract

Walnut (*Juglans regia* L.) is an oil-rich nut valued for its nutritional quality and characteristic flavor. Different thermal processing methods, including roasting, microwave treatment, and stir-frying, may shape walnut sensory perception by altering the composition and abundance of key aroma compounds and thereby influencing their differential interactions with human olfactory receptors. However, these relationships remain insufficiently understood. Here, walnut aroma under different thermal treatments was investigated using HS-SPME-GC–MS, sensory evaluation, electronic nose analysis, and high-throughput molecular docking. A total of 54 volatile compounds were identified, and thermal processing markedly promoted the formation of pyrazines, aldehydes, and some furans. Roasted samples showed the strongest nutty and roasted attributes and the highest acceptability. Correlation analysis indicated that pyrazines were closely associated with nutty and roasted notes, whereas some lipid oxidation-derived aldehydes and alcohols were related to fatty and green notes. Docking identified OR2AK2, OR11H2, and OR2G2 as candidate receptors.

## Graphical abstract

**Figure:**
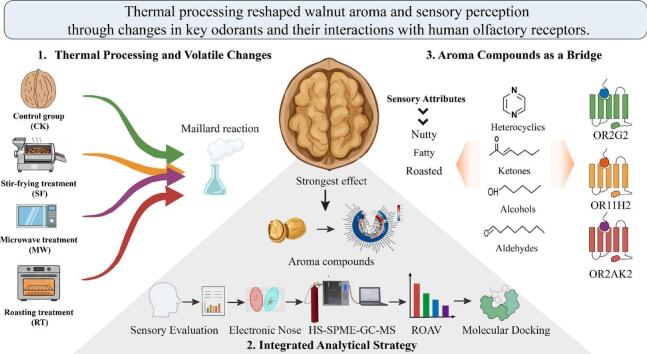

## 1 Introduction

Walnut (*Juglans regia* L.) belongs to the genus *Juglans* in the family Juglandaceae. As an important oil-producing woody crop, walnut is renowned worldwide for its high nutritional value and pleasant flavor. Walnuts are cultivated in over 50 countries and regions globally (Onwezen et al., 2021). They are rich in various nutrients, including unsaturated fatty acids, vitamin E, phenolic compounds, and proteins, which have made them highly favored by consumers worldwide (Alshahrani et al., 2022). In recent years, consumer demand for walnuts has become increasingly diverse, extending beyond nutritional quality to include flavor quality.

Raw walnuts usually have a mild aroma, with some undesirable notes such as green, astringent, and oily odors, which can limit their sensory acceptability to some extent. Appropriate thermal processing can effectively improve walnut flavor and serves as an important strategy for enhancing both edible quality and processing value (Peng et al., 2023). Thermal treatments, including roasting, microwave treatment, and stir-frying, can induce a series of internal reactions in walnuts, mainly lipid oxidation, the Maillard reaction, and Strecker degradation. These reactions generate a series of volatile flavor compounds (Franklin et al., 2018). Among them, the Maillard reaction is considered as a key pathway for the formation of volatile flavor compounds during thermal processing. This reaction is initiated by reducing sugars and amino compounds and proceeds through a series of degradation, dehydration, fragmentation, cyclization, and condensation reactions. It forms compounds including furans, pyrazines, pyrroles, and cyclic ketones, which contribute to typical roasted, nutty, and caramel-like aromas (Martins et al., 2000; Siegmund & Murkovic, 2004; Suri et al., 2022). In addition, Strecker degradation further promotes the formation of aldehydes and pyrazines, which are important aroma-active compounds (Martin & Ames, 2001; Yaylayan, 2003). Variations in heating conditions may also influence the composition and concentration of volatile compounds formed in plant oils (Park et al., 2011).

Among commonly used thermal pretreatment methods, microwave processing has attracted considerable attention because of its high heating efficiency, relatively uniform heating, and reduced thermal damage. It can also help preserve important nutrients and maintain product quality (Manzo et al., 2017). Ma et al. (2024) compared different thermal processing methods, including hot-air heating, microwave treatment, and stir-frying, and reported that thermal pretreatment not only significantly improved the oil yield and nutritional components of walnut oil but also helped retain more flavor compounds (Cao et al., 2024). Moreover, the content of pyrazines has been shown to increase with thermal treatment intensity; when walnuts were subjected to microwave processing at 600 W for more than 2 min, sensory panelists were able to perceive a distinct roasted aroma (Zhou et al., 2016). Kalogiouri et al. (2021) further reported that thermal pretreatment significantly increased the levels of seven pyrazine compounds, giving walnut oil a typical roasted flavor. Similarly, roasting and stir-frying are traditional but essential methods that significantly contribute to the nutty profile through distinct heat transfer mechanisms. Therefore, several commonly used thermal processing methods were selected to investigate their effects on walnut flavor characteristics and volatile compound formation.

Previous studies have investigated the effects of thermal processing on walnut flavor primarily from the perspectives of volatile compound composition and sensory characteristics. Different heating methods have varying effects on aroma formation due to differences in their heat transfer behavior and reaction pathways (Zhang et al., 2021). However, most existing studies have primarily focused on comparisons among processing methods or on the optimization of processing parameters. Systematic investigations into how key aroma compounds are formed under different thermal treatments and how these changes are associated with specific sensory attributes remain limited. Furthermore, although food aroma perception is ultimately mediated by interactions between odorants and olfactory receptors, the molecular basis of walnut aroma recognition has not yet been clearly elucidated.

Food aromas must be recognized by olfactory receptors before they can be perceived as sensory experiences. Humans possess approximately 400 functional olfactory receptors (Billesbølle et al., 2023). However, the currently known receptor–ligand pairing information remains limited. Previous studies have shown that some broadly tuned human olfactory receptors can recognize a wide range of key food-derived odor molecules. Meanwhile, molecular docking has emerged as a computational approach for simulating the interactions between small molecules and olfactory receptors. This technique enables researchers to analyze binding sites, interaction forces, and potential recognition mechanisms between odor molecules and olfactory receptors (Wang, Wang, et al., 2025). Therefore, integrating the screening of key aroma compounds with molecular docking analysis of olfactory receptors may help explain, from a molecular recognition perspective, why differences in chemical composition can ultimately lead to variations in sensory perception.

In this study, walnuts subjected to different thermal processing methods, including roasting, stir-frying, and microwave treatment, were used as the research materials to systematically investigate the composition and variation of volatile flavor compounds. Volatile compounds were first identified and quantified using HS-SPME-GC–MS, and key aroma compounds contributing significantly to the characteristic flavor of thermally processed walnuts were screened based on their ROAV values. Sensory evaluation was conducted to characterize the flavor attributes of walnuts under different processing conditions, allowing the relationship between key odorants and sensory attributes to be clarified. Furthermore, molecular docking analysis was employed to predict the potential interactions between key aroma compounds and human olfactory receptors (ORs), so as to further explain how thermal processing-induced changes in key odorants may influence sensory perception through differential receptor recognition. Through this integrated strategy, the present study aimed to clarify the relationships among thermal processing methods, key aroma compounds, sensory attributes, and olfactory receptor recognition in walnuts.

## 2 Materials and methods

### 2.1 Sample preparation

Walnut kernels (cultivar Xin 2) harvested in 2023 from Hotan, Xinjiang, China, were used in this study. To assess the effects of thermal processing on walnut flavor, three heat treatments were applied: roasting treatment (RT), microwave treatment (MW), stir-frying treatment (SF) and Untreated kernels (CK)served as the control.

The processing parameters were selected based on previous studies and preliminary trials. Although the temperature and treatment duration differed among roasting, microwave treatment, and stir-frying, these conditions were chosen to represent commonly used thermal processing methods for walnut kernels and to obtain a comparable degree of thermal processing among treatments. During preliminary trials, the processing conditions were adjusted with reference to the internal heating status of walnut kernels to avoid insufficient or excessive heating. For the RT, peeled walnut kernels were spread on a baking tray (approximately 8 cm thickness) and roasted in an oven at 130 °C for 20 min (Xu et al., 2023). For MW, peeled walnut kernels were evenly spread on a tray and heated at 560 W for 1 min (Peng et al., 2023). For the SF, a roasting machine was preheated to 130 °C, after which 500 g of peeled walnut kernels were added and stir-fried continuously for 20 min. Untreated walnut kernels from the same batch were used as the CK group. The CK samples were prepared at the same time as the thermally treated samples and were not exposed to room-temperature conditions for an extended period. After processing or preparation, all samples were cooled to room temperature, ground into powder using a mortar, sealed immediately, and stored under the same conditions before further analysis to minimize natural oxidation.

### 2.2 Sensory evaluation

A sensory panel was recruited from undergraduate and graduate students majoring in food science at China Agricultural University through a questionnaire survey. Candidates with smoking or drinking habits or respiratory diseases were excluded. A total of 35 candidates (12 males and 23 females) participated in the preliminary screening.

Selected candidates then received training on walnut aroma evaluation. During training, six aroma descriptors were introduced and defined: nutty, roasted, toasty, fatty, green, and burnt. Reference materials were provided for each attribute to improve consistency across panelists. After training, 24 panelists who could correctly recognize the aroma descriptors and showed relatively consistent scoring performance were selected for the formal sensory evaluation.

For the formal evaluation, coded walnut samples were presented to the panelists in a randomized order. Panelists evaluated the intensity of each aroma attribute and the overall acceptability of each sample according to the sensory evaluation sheet. Each sample was evaluated in triplicate. A 10 min break was scheduled between sessions to minimize olfactory fatigue. Mean values were used as the final sensory scores (Peng et al., 2023).

All sensory procedures involving human participants in this study were conducted in accordance with the Declaration of Helsinki or comparable ethical standards and were approved by the Ethics Committee for Human Research of China Agricultural University (Approval No. CAUHR-20240908). All participants provided informed consent before taking part in the sensory evaluation.

### 2.3 Electronic nose analysis

Electronic nose analysis was carried out with a Portable Electronic Nose Pen 3 system. For each measurement, 5.0 g of walnut sample was transferred to a 20 mL headspace vial and equilibrated for 10 min at room temperature. The operating conditions were as follows: sampling interval, 1 s; sensor cleaning time, 60 s; injection time, 5 s; flow rate, 400 mL/min; and total measurement time, 80 s. Signals recorded from 69 to 71 s were used for subsequent analysis. Each treatment was measured in triplicate (Booth et al., 2024).

### 2.4 Headspace solid-phase microextraction and gas chromatography–mass spectrometry analysis(HS-SPME-GC–MS)

Walnut kernels from each treatment were ground into powder and stored in sealed bags until analysis. For extraction, 2.0 g of walnut powder was placed in a 20 mL headspace vial, and 5 μL of o-dichlorobenzene in dichloromethane (0.0325 mg/mL) was added as the internal standard, followed by a magnetic stir bar. After equilibration at 60 °C for 20 min, volatile compounds were extracted using a conditioned DVB/CAR/PDMS fiber (50/30 μm, 2 cm) exposed to the headspace above the sample for 30 min at 60 °C. The fiber was then immediately inserted into the GC inlet and thermally desorbed at 250 °C for 8 min.

GC–MS analysis was carried out on an HP-5MS capillary column (30 m × 0.25 mm × 0.25 μm) using helium (>99.999%) as the carrier gas at a constant flow rate of 1.0 mL/min. The oven temperature was programmed from 50 °C (2 min) to 200 °C at 5 °C/min, then to 230 °C at 5 °C/min, followed by a 5 min hold. Mass spectra were recorded in electron ionization mode at 70 eV over an *m*/*z* range of 40–450.

### 2.5 Identification and quantification of volatile compounds

Volatile compounds were tentatively identified by comparison of their mass spectra with the NIST17 library and further verified by retention index (RI) values. A homologous series of n-alkanes (C7–C40) was analyzed under the same chromatographic conditions, and RI values were calculated from the retention times of the target compound and the adjacent n-alkanes. Compound identities were then confirmed by comparing the calculated RI values with those reported in the literature and relevant databases.

Quantification was performed using the internal standard method. The concentration of each analyte was calculated from the ratio of its peak area to that of the internal standard together with the known concentration of the internal standard. Therefore, these semi-quantitative data were mainly used to compare the relative abundance and treatment-induced changes of volatile compounds among different walnut samples.

### 2.6 Relative odor activity value (ROAV)

Odor activity values (OAV) were calculated as:

(1) $${\mathrm{OAV}}_{i}=\frac{C_{i}}{{\mathrm{OT}}_{i}}$$

and relative odor activity values (ROAV) were obtained by normalizing each OAV to the maximum value:

(2) $${\mathrm{ROAV}}_{i}=100\times \frac{{\mathrm{OAV}}_{i}}{{\mathrm{OAV}}_{\max}}$$

where $C_{i}$is the concentration of compound iderived from GC–MS quantification, $OT_{i}$is the odor threshold of compound *i, an*d $\mathrm{OA}V_{\max}$is the highest OAV among all detected compounds. Odor threshold values were obtained from Odor Thresholds (Gemert, 2011) and other published literature. When available, thresholds reported in oil-based matrices were preferentially used. For compounds without available oil-based thresholds, thresholds reported in aqueous, air, or other food-related matrices were used as alternatives. Because thresholds determined in different matrices may introduce uncertainty when applied to walnut samples, ROAV values were mainly used to compare the relative odor contributions of volatile compounds under the same calculation framework.

### 2.7 High-throughput molecular docking of key aroma compounds with ORs

Human olfactory receptor protein sequences were obtained from the Human Olfactory Data Explorer (HORDE) database. Their three-dimensional structures were predicted using AlphaFold3 on the basis of the corresponding amino acid sequences, and the putative active sites were determined accordingly. The quality of the AlphaFold3-predicted receptor models was evaluated based on the model confidence information generated by AlphaFold3 and the overall integrity of the seven-transmembrane architecture. To further examine the modeling procedure, a representative olfactory receptor with an available cryo-EM structure was modeled three times, and the predicted models were compared with the reported cryo-EM structure. Receptor models with reasonable overall folding and complete transmembrane structures were retained for subsequent molecular docking analysis. The predicted receptor models were then evaluated using SWISS-MODEL Tools, and Ramachandran plot analysis was further employed to examine the structural quality and stereochemical rationality of the models. Molecular docking was carried out using AutoDock Vina 1.2.7, and binding affinity (kcal/mol) was used as the indicator to determine the interaction between receptors and ligands (Trott & Olson, 2010). Binding interactions were analyzed using the Protein–Ligand Interaction Profiler (PLIP), and the corresponding binding conformations were visualized with UCSF ChimeraX (Zhao et al., 2025).

### 2.8 Statistical analysis

All data are presented as mean ± standard deviation from triplicate experiments. Statistical analyses were performed using SPSS 20.0 (IBM, Chicago, IL, USA). Significant differences among samples were determined by one-way ANOVA followed by Duncan's multiple range test at *p* < 0.05. Spearman correlation analysis was used to assess the relationships between key aroma compounds and sensory attributes, and principal component analysis was performed using Origin (OriginLab, Northampton, MA, USA).

## 3 Results and discussion

### 3.1 Sensory analysis

**Fig. 1:**
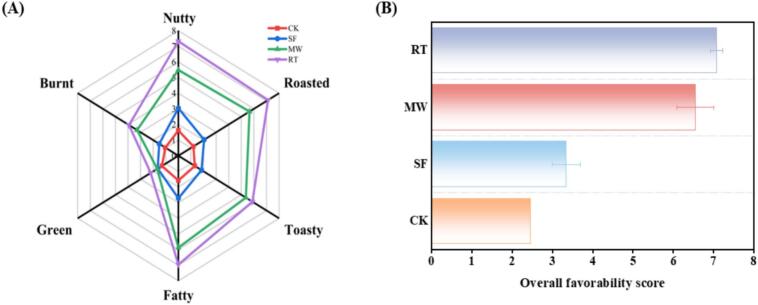
Sensory evaluation and preference scores of walnuts under different treatments: (A) radar plot of sensory attributes; (B) overall preference scores.

Different thermal processing methods significantly affected the sensory flavor characteristics of walnut samples (Fig. 1A). Compared with the untreated control samples, all three thermal treatments increased the intensity of nutty and roasted aromas in walnuts, indicating that heating promoted the formation of typical nutty flavor characteristics.

Among the thermal treatments, roasted samples received the highest scores for most sensory attributes, with more pronounced nutty and roasted notes, stronger fatty aroma, and greater overall flavor intensity. Microwave treatment also enhanced these sensory attributes, but to a lesser extent. Stir-frying produced no significant difference in flavor intensity.

Preference scores showed a trend similar to that of sensory intensity (Fig. 1B). Roasted samples had the highest overall acceptability, followed by microwave-treated, stir-fried, and untreated samples (RT > MW > SF > CK). These results indicate that thermal processing improved walnut flavor quality and sensory acceptance, with roasting showing the strongest effect.

### 3.2 Electronic nose analysis

To further evaluate the effects of thermal processing on the overall odor profile of walnuts, an electronic nose was used to analyze the volatile odor characteristics of the samples. All thermally treated walnut kernels produced clear responses across multiple sensors, although the intensity varied among treatments. The overall response patterns were broadly similar, suggesting that the samples shared some common volatile features. At the same time, thermal processing appeared to promote the formation or release of specific volatile compounds, leading to changes in walnut aroma.

**Fig. 2:**
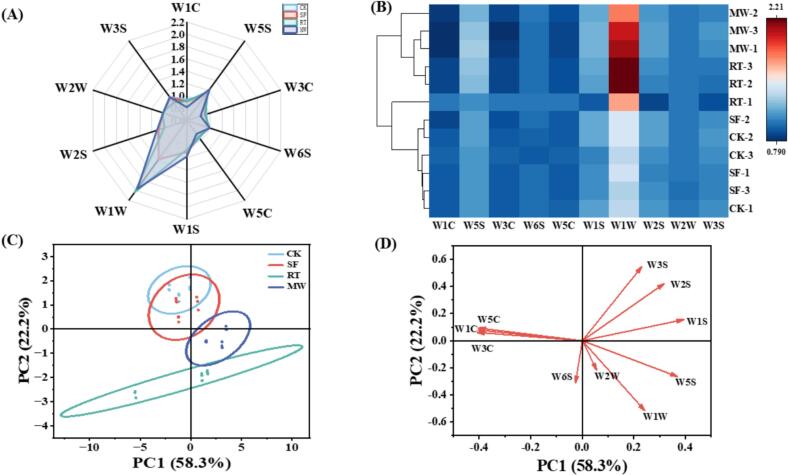
Electronic nose analysis of walnuts under different treatments: (A) radar plot of electronic nose sensor responses; (B) hierarchical clustering heatmap of electronic nose sensor responses; (C) PCA score plot based on electronic nose data; (D) PCA loading plot of electronic nose sensors.

The radar plot (Fig. 2A) indicated differences in sensor response patterns among treatments. In particular, the W1W sensor gave the strongest responses for roasted and microwave-treated samples. Because W1W is sensitive to sulfur-containing compounds, pyrazines, and terpenes, this result suggests that these compounds were enhanced by thermal processing. The W5S, W1S, and W2S sensors also showed relatively strong responses in some samples, indicating treatment-related differences in nitrogen oxides, alkanes, alcohols, and other aroma-active compounds. Together with the sensory results, these findings suggest that thermal processing promoted the formation of multiple classes of volatiles and contributed to a richer and more complex walnut aroma.

The hierarchical clustering heatmap further highlighted differences in electronic nose response patterns among treatments (Fig. 2B). Replicates from the same treatment clustered closely, indicating good measurement repeatability. Roasted and microwave-treated samples were grouped more closely than the other treatments, suggesting that these two treatments produced more similar odor profiles. In contrast, the control and stir-fried samples were separated to different degrees. These results indicate that thermal processing changed the overall volatile profile of walnut kernels, although the extent of change depended on the treatment.

Principal component analysis also revealed clear differences in odor characteristics among treatments (Fig. 2C). The first two principal components explained 58.3% and 22.2% of the total variance, respectively, indicating that they captured most of the odor information detected by the electronic nose. The score plot showed clear separation among treatment groups, with tight clustering within each group, confirming that different thermal processing methods significantly affected the overall odor profile of walnuts. The loading plot (Fig. 2D) showed that W3S, W2S, and W1S contributed strongly to PC1 and/or PC2, while W1W and W5S also played important roles in sample discrimination. By contrast, W1C, W5C, and W3C were located mainly on the negative side of PC1 and contributed less to treatment separation. Overall, these results suggest that sensors associated with sulfur compounds, pyrazines, alcohols, aldehydes, ketones, methyl compounds, and nitrogen oxides were the main contributors to differences among walnut samples.

### 3.3 Composition analysis of volatile compounds

HS-SPME-GC–MS was used to analyze the volatile compounds in walnut samples subjected to different treatments. In total, 54 compounds were identified, including aldehydes, alcohols, ketones, acids, heterocyclic compounds, aromatic compounds, and a small number of other compounds.

**Fig. 3:**
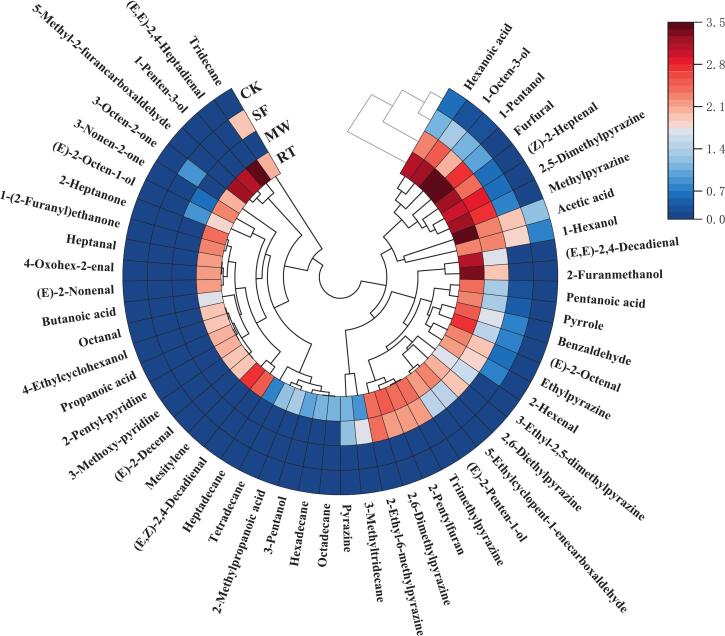
Cluster heatmap of volatile compounds in walnuts under different treatments.

The number of volatile compounds varied greatly among treatments. Roasted samples contained 54 volatile compounds, followed by microwave-treated samples with 25 and stir-fried samples with 19, whereas only 11 compounds were detected in the untreated control(supplementary Table 1). The control sample had generally low levels of volatile compounds, whereas many compounds increased after thermal processing, with the largest changes observed in the roasted samples (Fig. 3). Roasted and microwave-treated samples clustered together, indicating relatively similar overall volatile profiles. In contrast, the control sample formed a separate cluster, while the stir-fried sample showed an intermediate profile. These results indicate that thermal processing changed not only the abundance of volatile compounds but also the overall aroma profile of walnuts (Zhang et al., 2026).

**Fig. 4:**
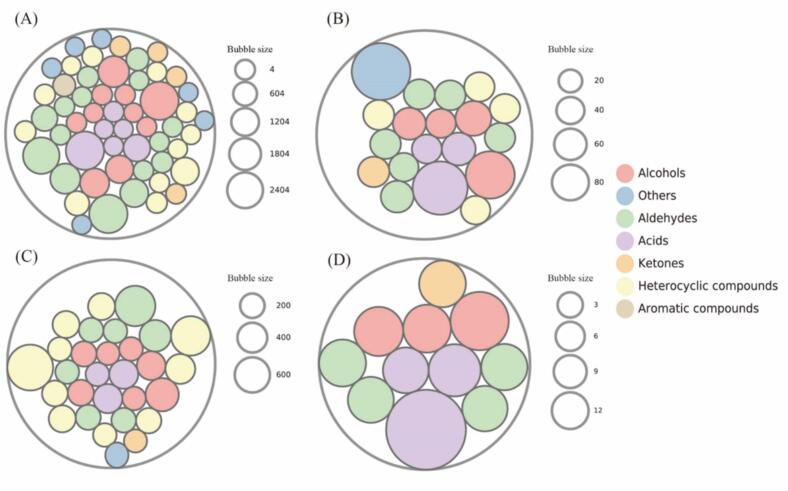
Bubble plots of volatile compound classes in walnuts under different treatments: (A) RT; (B) SF; (C) MW; (D) CK.

The circular stacked plot provided additional information on the distribution of volatile classes under different treatments (Fig. 4). Aldehydes, alcohols, and heterocyclic compounds were the dominant groups, although their relative proportions varied markedly among treatments. The control sample contained fewer volatile compounds and consisted mainly of low levels of aldehydes and alcohols, indicating a relatively simple aroma profile. Compared with the control, stir-fried samples showed clear increases in both the number and abundance of volatile compounds, especially aldehydes, alcohols, and acids, suggesting that stir-frying promoted the formation of multiple volatile compounds. Microwave treatment also increased the complexity of the volatile composition, particularly by enhancing the relative abundance of heterocyclic compounds, aldehydes, and alcohols. In roasted samples, the proportions of heterocyclic compounds and aldehydes increased further, indicating that roasting was more favorable for the formation of characteristic aroma compounds and produced a richer overall aroma.

Among the different classes of volatiles, heterocyclic compounds showed the most pronounced changes after thermal treatment and appeared to be an important basis for the typical roasted and nutty aroma of walnuts. These compounds mainly included pyrazines, furans, and pyrroles, with pyrazines showing the largest increase. In particular, 2-ethyl-6-methylpyrazine, 2,6-diethylpyrazine, ethylpyrazine, and 3-ethyl-2,5-dimethylpyrazine increased markedly in the heat-treated samples. These compounds are generally formed through the Maillard reaction and Strecker degradation. In these reactions, reducing sugars react with amino acids to form reactive carbonyl intermediates, which then undergo condensation, cyclization, and dehydrogenation to produce nitrogen-containing heterocyclic compounds. The higher abundance of pyrazines in the heat-treated samples, especially in roasted samples, may be associated with Maillard-related reactions and Strecker degradation under the applied processing conditions, thereby contributing roasted, nutty, and fatty notes to the samples (Zhu et al., 2026).

Aldehydes are also important components of the walnut flavor system, and their formation is mainly related to the oxidative cleavage of unsaturated fatty acids. The aldehydes detected in this study included 2-hexenal, octanal, (*E*)-2-octenal, (*E*)-2-decenal, (*E,E)*-2,4-heptadienal, and (*E,E*)-2,4-decadienal. These compounds usually have low odor thresholds and can impart green, fatty, and fruity notes to the samples. As thermal treatment proceeded, lipid oxidation intensified, which likely promoted the formation of these aldehydes (Shahidi & Hossain, 2022).

Alcohols, including 1-octen-3-ol, 1-hexanol, (*E*)-2-penten-1-ol, and (*E*)-2-octen-1-ol, were also detected at appreciable levels. These compounds were mainly derived from fatty acid oxidation or from the further reduction of aldehydes. Unlike the strong roasted aroma imparted by heterocyclic compounds, alcohols contribute more green, mushroom-like, or floral notes, and mainly serve to modify and enrich the aroma layers in the overall aroma profile (Yuan et al., 2025).

Overall, under the processing conditions used in this study, thermal processing markedly changed the number, abundance, and composition of volatile compounds in walnuts. These changes may be associated with multiple reaction pathways, including Maillard-related reactions, Strecker degradation, and lipid oxidation-related processes. Among the tested treatments, roasting showed the strongest effect, particularly by promoting the formation of key heterocyclic compounds and aldehydes, thereby producing a more intense and complex walnut aroma.

### 3.4 Key aroma compounds and their correlation with sensory attributes

Relative odor activity value (ROAV) was used to estimate the contribution of individual odorants to the overall aroma of the sample, while also accounting for matrix-related effects. Generally, when ROAV >1, the compound is regarded as a key aroma compound contributing significantly to the overall aroma of the sample (Zhu et al., 2020).

Based on the ROAV results, several key aroma compounds were identified in Table 1, including (*E,E*)-2,4-decadienal, (*E*)-2-decenal, (*E*)-2-octen-1-ol, (*E,E*)-2,4-heptadienal, 3-ethyl-2,5-dimethylpyrazine, 2,6-diethylpyrazine, 1-octen-3-ol, 3-octen-2-one, 2-ethyl-6-methylpyrazine, 1-hexanol, ethylpyrazine, (*Z*)-2-heptenal, octanal, and pyrazine. Among these compounds, pyrazines accounted for a substantial proportion, while several aldehydes also showed relatively high ROAV values. These findings indicate that the characteristic aroma of the samples was mainly driven by a limited number of highly potent odorants, rather than by all detected volatiles equally, with pyrazines and aldehydes representing the major contributors.

**Table 1:** ROAV>1 of volatile compounds in different thermal treatments of walnuts.⁎

Compound	Odor description	Odor threshold (μg/kg)	ROAV RT	MW	SF	CK
(*E,E*)-2,4-Decadienal	citrus, fatty	0.07	29.946	1.760	4.119	N.D.
(*E*)-2-Decenal	citrus, fatty	0.03	5.239	N.D.	N.D.	N.D.
*(E*)-2-Octen-1-ol	green, mushroom	0.075	1.470	0.176	N.D.	N.D.
(*E,E*)-2,4-Heptadienal	green, fatty	0.1	59.666	N.D.	N.D.	N.D.
3-Ethyl-2,5-dimethylpyrazine	nutty, roasted	0.084	1.183	0.867	N.D.	N.D.
2,6-Diethylpyrazine	roasted, nutty	0.05	4.804	3.482	3.804	N.D.
1-Octen-3-ol	mushroom, earthy	0.153	20.513	4.882	69.704	43.534
3-Octen-2-one	mushroom, earthy	0.034	6.454	N.D.	100.000	100.000
2-Ethyl-6-methylpyrazine	roasted, nutty	0.005	100.000	100.000	N.D.	N.D.
1-Hexanol	green, floral	1	0.390	0.423	31.774	19.565
Ethylpyrazine	nutty, roasted	0.25	1.661	0.830	6.514	N.D.
(*Z*)-2-Heptenal	fruity, fatty	1.93	1.467	0.324	0.955	1.131
Octanal	fatty, citrus, honey	0.0034	52.203	N.D.	N.D.	N.D.
Pyrazine	Nutty, roasted	0.02	1.344	1.992	N.D.	N.D.

⁎ Note: odor threshold values were obtained from Odor Thresholds (Gemert, 2011) and other published literature. N.D. indicates not detected.

**Fig. 5:**
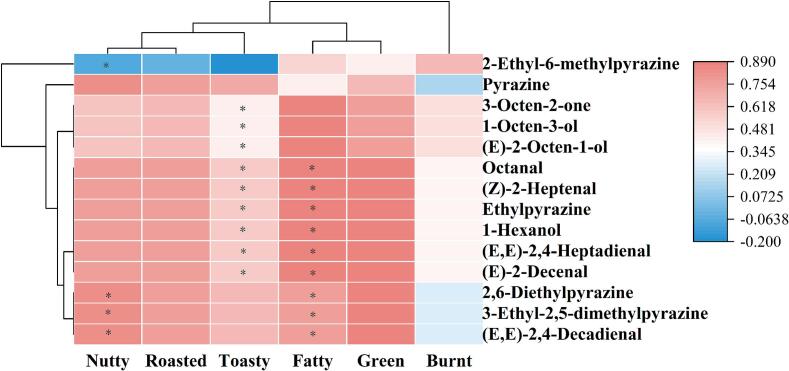
Correlation heatmap of key aroma compounds and sensory attributes in walnuts.

To further examine the relationships between key aroma compounds and sensory attributes, Spearman correlation analysis was performed (Fig. 5). This analysis focused on roasted and microwave-treated samples because these two treatments showed the strongest increases in nutty and roasted scores compared with the control and stir-fried samples (Fig. 1). Focusing on these treatments reduced the influence of background differences and allowed clearer identification of the compounds most closely associated with desirable walnut aroma.

Most key aroma compounds showed significant correlations with sensory attributes, although the patterns differed among compound classes. Nutty, roasted, and toasted attributes were strongly positively correlated with one another and were also significantly associated with several key aroma compounds formed during thermal processing. In particular, 2,6-diethylpyrazine, 3-ethyl-2,5-dimethylpyrazine, and (*E,E*)-2,4-decadienal showed relatively high correlation coefficients with these typical roasted sensory attributes, suggesting that they are important contributors to the nutty and roasted aroma of walnuts.

By contrast, several compounds associated with lipid oxidation, including octanal, (*E*)-2-decenal, (*Z*)-2-heptenal, and 1-hexanol, were more closely related to fatty and green sensory attributes. These results show that different groups of aroma compounds contributed differently to walnut flavor, with thermally generated pyrazines playing a central role in roasted and nutty sensory attributes, while lipid oxidation products were more important contributors to fatty and green attributes.

### 3.5 High-throughput molecular docking analysis of key aroma compounds with olfactory receptors

**Fig. 6:**
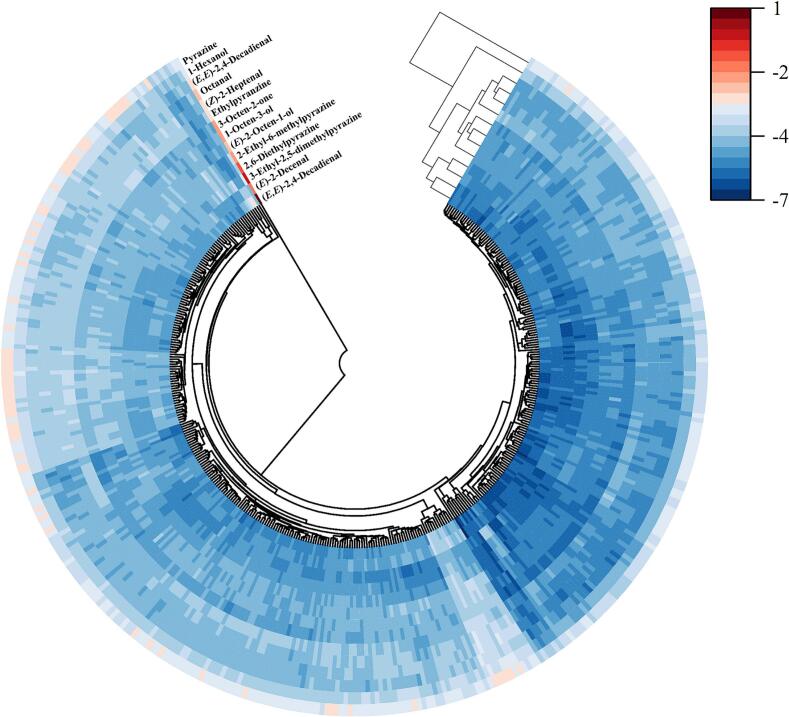
Circular clustered heatmap of molecular docking binding energies between key aroma compounds and human olfactory receptors.

To further interpret the sensory differences induced by thermal processing from a molecular recognition perspective, high-throughput molecular docking was performed between the screened key aroma compounds and human olfactory receptors. It has been reported that aroma compounds are recognized through approximately 400 olfactory receptors (ORs) in humans. Protein sequences of 391 class II human olfactory receptors were retrieved from the Human Olfactory Receptor Data Explorer (HORDE) database. These sequences were subsequently used as input for AlphaFold3 to generate the corresponding three-dimensional receptor models. Using this strategy, three-dimensional structural models of relatively good quality were ultimately generated for all 391 olfactory receptors, providing a structural foundation for subsequent molecular docking analyses. Molecular docking analysis was conducted between the key aroma compounds screened by ROAV and 391 olfactory receptors. As shown in Fig. 6 and supplementary table 2, the circular clustered heatmap revealed substantial variation in binding energies among different odorant–receptor pairs, indicating clear receptor-binding selectivity of the key aroma compounds. The results suggested receptor-dependent differences in predicted binding tendencies between key aroma compounds and olfactory receptors, and some aroma-active compounds showed relatively favorable predicted interactions with multiple receptors.

After classifying the key aroma compounds according to their chemical structural characteristics, receptors were ranked based on their predicted binding energies within each compound category. The top ten receptors with the lowest binding energies were selected for each category as dominant candidate receptors and were then subjected to intersection analysis. This selection was based on relative binding energy ranking rather than a fixed binding energy threshold, because binding affinities may differ among different odorant classes. The results revealed that OR2AK2, OR11H2, and OR2G2 repeatedly appeared among the dominant receptors for different categories of aroma compounds, suggesting that these receptors may have relatively broad predicted binding profiles toward multiple classes of key walnut aroma compounds. Therefore, these receptors may be regarded as candidate core olfactory receptors potentially associated with the predicted binding of key walnut aroma compounds.

Differences in amino acid composition give rise to functional diversity among olfactory receptors, enabling them to selectively respond to a wide range of odor molecules and thereby contributing fundamentally to odor discrimination (Mei et al., 2023). As summarized in Table 2, the key aroma compounds showed distinct receptor-binding preferences. Their binding energies with different olfactory receptors ranged from −6.144 to −3.864 kcal/mol, suggesting generally favorable docking scores among the selected odorant–receptor pairs. Importantly, the same odorant exhibited distinct binding affinities and interacted with different binding regions among individual ORs.

**Table 2:** Summary of the binding energy between olfactory receptors and ligands.

Ligands	Binding energy (kcal/mol)		
	OR11H2	OR2G2	OR2AK2
(*E,E*)-2,4-Decadienal	−5.856	−6.004	−6.061
(*E,E*)-2,4-Heptadienal	−5.307	−4.949	−5.417
(*Z*)-2-Heptenal	−5.429	−5.254	−5.494
Octanal	−5.463	−5.592	−5.23
(*E*)-2-Decenal	−5.57	−5.846	−5.772
(*E*)-2-Octen-1-ol	−5.366	−6.04	−5.756
1- Hexanol	−4.981	−4.922	−5.089
1-Octen-3-ol	−5.888	−5.423	−5.378
3-Octen-2-one	−5.981	−5.786	−5.922
2-Ethyl-6-methylpyrazine	−5.791	−5.948	−5.65
Ethylpyrazine	−5.639	−5.383	−5.283
Pyrazine	−3.901	−3.864	−4.2
3-Ethyl-2,5-dimethylpyrazine	−6.05	−5.862	−5.324
2,6-Diethylpyrazine	−6.144	−6.239	−5.672

Among the screened candidate receptors, key aroma compounds with different structural characteristics all exhibited strong binding tendencies. In particular, pyrazine compounds generally showed lower binding energies, indicating stronger receptor-binding affinities. For example, the binding energy between 2,6-diethylpyrazine and OR2G2 reached the lowest value of −6.239 kcal/mol, while 3-ethyl-2,5-dimethylpyrazine and OR11H2 exhibited a binding energy of −6.050 kcal/mol, suggesting that these typical roasted-flavor compounds may interact with the candidate receptors.

For aldehyde compounds, (*E,E*)-2,4-decadienal showed a binding energy of −6.061 kcal/mol with OR2AK2, also indicating strong binding affinity. In addition, some aliphatic alcohol compounds exhibited notable binding advantages; for example, (*E*)-2-octen-1-ol and OR2G2 showed a binding energy of −6.040 kcal/mol.

**Fig. 7:**
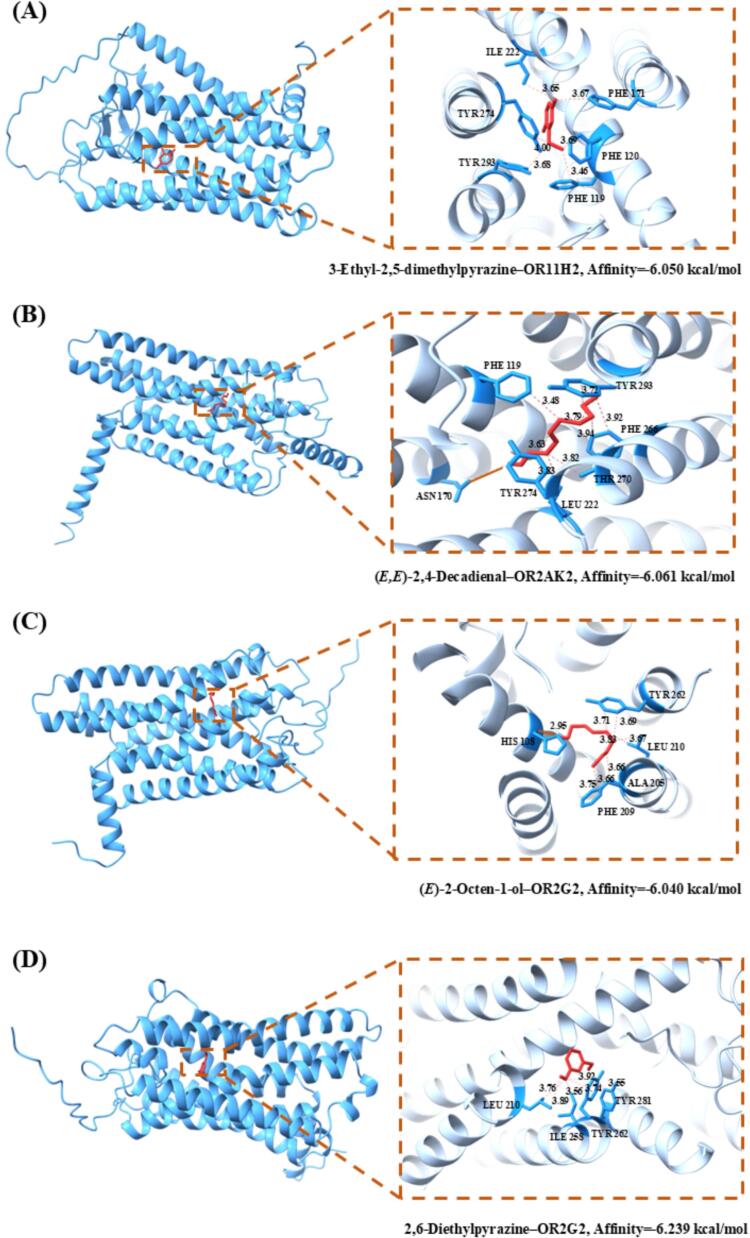
Molecular docking conformations and binding interactions of representative key aroma compounds with candidate olfactory receptors: (A) 3-ethyl-2,5-dimethylpyrazine–OR11H2; (B) (*E,E*)-2,4-decadienal–OR2AK2; (C) (*E*)-2-octen-1-ol–OR2G2; (D) 2,6-diethylpyrazine–OR2G2.

To further explore the predicted binding sites and intermolecular interactions, representative compounds and their dominant receptors were selected for interaction visualization analysis. The results showed that 3-ethyl-2,5-dimethylpyrazine mainly interacted with OR11H2 through hydrophobic interactions, involving residues PHE119 (3.46 Å), PHE120 (3.69 Å), PHE171 (3.67 Å), ILE222 (3.65 Å), TYR274 (4.00 Å), and TYR293 (3.68 Å) (Fig. 7A).

For (*E,E*)-2,4-decadienal and OR2AK2, in addition to hydrophobic interactions, one hydrogen bond was formed, in which ASN170 participated in the hydrogen bonding interaction, while residues PHE119 (3.48 Å), LEU222 (3.83 Å), PHE266 (3.92 Å, 3.94 Å), THR270 (3.82 Å), TYR274 (3.63 Å), and TYR293 (3.79 Å, 3.72 Å) contributed to hydrophobic interactions(Fig. 7B).

For (*E*)-2-octen-1-ol and OR2G2, a hydrogen bond mediated by HIS108 was formed, along with hydrophobic interactions involving residues ALA205 (3.66 Å), PHE209 (3.66 Å, 3.75 Å), LEU210 (3.89 Å, 3.67 Å), and TYR262 (3.71 Å, 3.69 Å) (Fig. 7C).

In contrast, the binding between 2,6-diethylpyrazine and OR2G2 mainly relied on hydrophobic interactions involving residues LEU210 (3.76 Å, 3.89 Å), ILE258 (3.56 Å), TYR262 (3.92 Å, 3.74 Å), and TYR281 (3.55 Å) (Fig. 7D).

Although many compounds formed only one or two hydrogen bonds with the olfactory receptors, they still exhibited extensive hydrophobic contacts with surrounding amino acid residues, suggesting that hydrophobic interactions are a major driving force for ligand recognition and complex stabilization (Chen et al., 2026). This was particularly evident for pyrazine compounds, whose aromatic heterocyclic structures favor stable packing within hydrophobic binding pockets and interactions with residues such as PHE, LEU, ILE, and TYR. Therefore, besides hydrogen bonding, hydrophobic interactions play a crucial role in the binding of walnut aroma compounds to candidate olfactory receptors and may explain the relatively low binding energies observed for several roasted-flavor pyrazines. These results suggest that pyrazine compounds showed favorable predicted binding tendencies toward these candidate receptors. However, whether these compounds can activate the corresponding receptors requires further functional validation. Wen et al. also reported, in their study on the molecular recognition mechanism of hOR9Q2 toward 4-methylphenol, that hydrophobic interactions and π‑sulfur interactions were the major driving forces for stable binding, while site-directed mutagenesis confirmed that key residues in the TM3, TM5, and TM6 domains, such as Phe, Leu, and Tyr, were crucial for ligand recognition. In the present study, the key residues identified by PLIP analysis, including PHE119, LEU210, and TYR262, were highly consistent with these findings, suggesting that aromatic and hydrophobic residues may represent conserved features of olfactory receptor ligand-binding pockets (Wen et al., 2025).

### 3.6 Analysis of receptor recognition of characteristic walnut aroma based on sensory correlation and high-throughput molecular docking

Different thermal processing methods significantly altered the composition and relative abundance of key aroma compounds in walnuts, and these changes may further influence sensory perception through differential interactions with human olfactory receptors. Combined analysis of volatile compounds and ROAV results showed that pyrazines, aldehydes, and some furans increased markedly after thermal processing, with the most pronounced changes observed in roasted and microwave-treated samples. These findings were consistent with the sensory evaluation results, in which thermally processed samples exhibited stronger nutty and roasted attributes, with roasting producing the greatest enhancement. This suggests that thermal processing shapes walnut flavor by regulating the formation of key aroma compounds.

Correlation analysis further showed that different classes of key aroma compounds contributed differently to sensory attributes. Pyrazines were more strongly and positively associated with typical heat-induced sensory attributes, particularly nutty and roasted attributes, whereas some aldehydes and alcohols derived from lipid oxidation were more closely related to fatty and green attributes. These results indicate that thermal processing does not simply change the total amount of volatile compounds, but more importantly alters the combination of key aroma compounds with distinct sensory contributions, thereby leading to differences in the sensory profiles of walnuts subjected to different treatments.

High-throughput molecular docking provided preliminary computational clues for understanding the potential molecular recognition of key aroma compounds by olfactory receptors. The predicted differences in odorant–receptor binding behaviors suggest that thermal processing-induced changes in aroma composition may be associated with different receptor-binding patterns. Binding energy differences between odorant–receptor pairs can be explained mainly by receptor heterogeneity, ligand structure, and spatial compatibility between the interacting molecules. Together, these factors determine binding strength and may ultimately affect aroma perception. The different binding behaviors of key aroma compounds are likely to generate distinct olfactory signals, and the integration of these signals may contribute to the characteristic and complex aroma of walnuts (Zhu et al., 2026). The docking results also showed clear differences in receptor-binding preferences among key aroma compounds, suggesting that thermal processing-induced changes in aroma composition may be translated into different receptor recognition patterns.

Among the thermally enhanced compounds, pyrazines appeared to play a particularly important role in roasted and nutty walnut aroma. In particular, 2,6-diethylpyrazine and 3-ethyl-2,5-dimethylpyrazine were significantly positively correlated with the nutty attribute and showed strong binding to candidate receptors such as OR2G2 and OR11H2, mainly through hydrophobic interactions. Among them, 2,6-diethylpyrazine showed the lowest binding energy with OR2G2 (−6.239 kcal/mol) and also strong binding to OR11H2 (−6.144 kcal/mol). Similarly, 3-ethyl-2,5-dimethylpyrazine showed binding energies of −6.050 and − 5.862 kcal/mol with OR11H2 and OR2G2, respectively, while ethylpyrazine also bound strongly to OR2G2 (−5.838 kcal/mol). These results suggest that OR2G2 and OR11H2 may be candidate receptor-binding targets potentially associated with roasted and nutty walnut aroma compounds. Notably, unsubstituted pyrazine, which has the simplest structure, showed negative correlations with nutty and roasted attributes in the heatmap and also exhibited the weakest binding energies in the docking analysis. This finding indicates that alkyl substitution is important for receptor recognition of pyrazine compounds. Ethyl- and dimethyl-substituted pyrazines showed markedly stronger binding affinities, suggesting that alkyl substitution may increase hydrophobicity and improve the fit of the ligand within the hydrophobic receptor pocket, thereby enhancing binding affinity. This interpretation is consistent with the findings of Wang et al., who reported that structural features of flavor compounds, including chain length and functional group position, significantly influence their binding ability toward proteins (Wang, Zhang, et al., 2025).

In contrast, some aldehydes and alcohols associated with fatty and green attributes showed different binding modes. In addition to hydrophobic interactions, (*E,E)*-2,4-decadienal formed a hydrogen bond with ASN170, and (*E*)-2-octen-1-ol formed a hydrogen bond with HIS108. These results suggest that different sensory attributes may be associated with different molecular recognition mechanisms. This interpretation is consistent with the findings of Ben et al., who reported that vanillin binding to mammalian olfactory receptors involved both van der Waals forces and hydrogen bonding, which together contributed to complex stability.(Ben Khemis et al., 2025).

Overall, the influence of thermal processing on walnut flavor was reflected not only in changes in the composition and abundance of key aroma compounds, but also in differences in the ways these compounds interacted with olfactory receptors. In other words, thermal processing may influence walnut flavor quality by reshaping the continuous process of key aroma compound formation, receptor recognition, and sensory expression. These results provide molecular-level preliminary computational insight into understanding how thermal processing regulates walnut aroma formation and perception, and offer a new perspective for further investigating flavor quality formation in nut-based foods from the viewpoint of olfactory receptor recognition.

## 4 Conclusion

Different thermal processing methods significantly affected the aroma characteristics of walnut kernels by altering the composition and relative abundance of volatile compounds. Compared with the untreated samples, all three thermal treatments enhanced the nutty and roasted attributes of walnuts, among which roasting showed the most pronounced effect on flavor improvement and achieved the highest overall sensory acceptability. Sensory evaluation and electronic nose analysis confirmed significant differences in flavor characteristics among the differently treated samples, with roasted walnuts showing the highest acceptability. Volatile analysis further demonstrated that roasting generated the greatest variety of volatile compounds and was particularly favorable for the formation of heterocyclic compounds and aldehydes, thereby contributing to a richer and more complex aroma profile. ROAV analysis further confirmed that pyrazines, aldehydes were the major contributors to the characteristic aroma of thermally processed walnuts. Among them, 2,6-diethylpyrazine, 3-ethyl-2,5-dimethylpyrazine, and (*E,E*)-2,4-decadienal were identified as important compounds closely associated with roasted and nutty attributes, whereas some lipid oxidation-derived volatiles were more strongly related to fatty and green notes. Molecular docking analysis indicated receptor-dependent differences in the binding behaviors of key aroma compounds toward human olfactory receptors, with OR2G2 and OR11H2 suggested as candidate receptors potentially associated with the recognition of roasted and nutty aroma compounds. By integrating chemical analysis, sensory science, and computational biology, this study provides a new framework for understanding flavor perception at the molecular level. These findings offer a theoretical basis for optimizing thermal processing conditions to improve walnut flavor quality and may also provide guidance for related product development.

## CRediT authorship contribution statement

**Yishen Cheng:** Writing – original draft, Data curation, Conceptualization. **Shanxing Gao:** Software, Methodology, Investigation. **Lei Zhang:** Supervision, Funding acquisition. **Chenyan Lv:** Visualization, Validation, Project administration. **Jiachen Zang:** Writing – review & editing, Funding acquisition.

## Fundings

This work was supported by National Key R & D Program of China (2024YFF1106603) and Central Government Guidance Fund for Local Sci-Tech Development (ZYYD2025QY09).

## Declaration of competing interest

The authors declare that they have no known competing financial interests or personal relationships that could have appeared to influence the work reported in this paper.

## Data Availability

Data will be made available on request.
