# Supplementary material for: Formation of characteristic aroma compounds in walnut kernels during thermal processing and their potential recognition by human olfactory receptors
Source: Food Chem X. 2026 Jul 9;37:104142. doi: 10.1016/j.fochx.2026.104142 (PMC13380225; doi:10.1016/j.fochx.2026.104142)
Supplement: Supplementary file 1 — Supplementary material 1 [file mmc1.docx]

**Table 1. Concentrations of Volatile Compounds in Walnut Samples under Different Thermal Processing Treatments**

| Compound | Concentration(μg/kg)  RT | | SF | MW | CK | IDc |
| --- | --- | --- | --- | --- | --- | --- |
| (E)-2-Decenal | 77.88±21.99b | | N.D. | N.D. | N.D. | MS/RI/ |
| (E)-2-Nonenal | 138.52±36.26b | | N.D. | N.D. | N.D. | MS/RI/ |
| (E)-2-Octen-1-ol | 54.65±14.17b | | N.D. | 5.67±0.32ab | N.D. | MS/RI/ |
| (E)-2-Octenal | 544.26±133.01a | | 4.01±1.38b | 29.55±0.59ab | 41.02±40.6ab | MS/RI/ |
| (E)-2-Penten-1-ol | 155.47±40.55b | | N.D. | 24.76±4.7ab | N.D. | MS/RI/ |
| (E,E)-2,4-Decadienal | 1038.76±298.49b | | 0.52±0.52ab | 52.93±3.99ab | N.D. | MS/RI/ |
| (E,E)-2,4-Heptadienal | 2956.71±689.26b | | N.D. | N.D. | N.D. | MS/RI/ |
| (E,Z)-2,4-Decadienal | 322.48±92.58b | | N.D. | N.D. | N.D. | MS/RI/ |
| (Z)-2-Heptenal | 1403.21±339.59b | | 3.31±0.9ab | N.D. | 0.69±0.1ab | MS/RI/ |
| 1-(2-Furanyl)ethanone | 201.26±39.26b | | N.D. | N.D. | N.D. | MS/RI/ |
| 1-Hexanol | 193.29±52.44b | | 57.17±3.49ab | N.D. | 6.38±1.06ab | MS/RI/ |
| 1-Pentanol | 2944.19±687.39b | | 10.53±0.82ab | 98.5±21.78ab | 1.64±0.28a | MS/RI/ |
| 1-Penten-3-ol | 1337.99±334.29b | | N.D. | N.D. | N.D. | MS/RI/ |
| 2,5-Dimethylpyrazine | 830.16±233.63b | | 2.87±0.58ab | 713.19±67.19b | N.D. | MS/RI/ |
| 2,6-Diethylpyrazine | 119.03±30.91b | | 0.34±0.34ab | 74.82±0.44ab | N.D. | MS/RI/ |
| 2,6-Dimethylpyrazine | 339.27±88.37b | | N.D. | 126.11±8.14ab | N.D. | MS/RI/ |
| 2-Ethyl-6-methylpyrazine | 247.77±68.74a | | N.D. | 214.88±9.05a | N.D. | MS/RI/ |
| 2-Furanmethanol | 1862.24±396.38b | | N.D. | 73.27±4.67ab | N.D. | MS/RI/ |
| 2-Heptanone | 222.32±124.46b | | N.D. | N.D. | N.D. | MS/RI/ |
| 2-Hexenal | 157.15±45.68b | | 4.27±2.82ab | 67.2±11.93ab | 1.15±0.13a | MS/RI/ |
| 2-Methylpropanoic acid | | 18.44±3.82b | N.D. | N.D. | N.D. | MS/RI/ |
| 2-Pentylfuran | | 255.89±68.53b | N.D. | 125.52±2.82ab | N.D. | MS/RI/ |
| 3-Ethyl-2,5-dimethylpyrazine | | 49.26±13.47b | N.D. | 31.29±1.23ab | N.D. | MS/RI/ |
| 3-Methyltridecane | | 5.66±1.79b | N.D. | N.D. | N.D. | MS/RI/ |
| 3-Nonen-2-one | | 163.33±43.94b | N.D. | 3.58±0.12ab | N.D. | MS/RI/ |
| 3-Octen-2-one | | 108.74±24.88b | 6.12±2.34ab | 28.41±1.47ab | 0.69±0.23a | MS/RI/ |
| 3-Pentanol | | 8.56±2.61b | N.D. | N.D. | N.D. | MS/RI/ |
| 4-Ethylcyclohexanol | | 90.77±23.66b | N.D. | N.D. | N.D. | MS/RI/ |
| 4-Oxohex-2-enal | | 153.24±36.86b | N.D. | N.D. | N.D. | MS/RI/ |
| 5-Ethylcyclopent-1-enecarboxaldehyde | | 171.06±42.2b | N.D. | N.D. | N.D. | MS/RI/ |
| 5-Methyl-2-furancarboxaldehyde | | 1700.57±351.32b | N.D. | N.D. | N.D. | MS/RI/ |
| Acetic acid | | 2954.82±535.36b | 74.86±19.48ab | 188.88±7.99ab | 6.94±6.55a | MS/RI/ |
| Benzaldehyde | | 325.89±90.69b | 4.7±0.43ab | 50.01±6.45ab | 0.43±0.3a | MS/RI/ |
| Butanoic acid | | 45.68±8.56b | N.D. | N.D. | N.D. | MS/RI/ |
| Ethylpyrazine | | 205.8±56.81b | 2.93±0.87ab | N.D. | N.D. | MS/RI/ |
| Furfural | | 2670.37±573.36b | 9.88±1.05ab | 541.16±71.6ab | N.D. | MS/RI/ |
| Heptadecane | | 4.67±2.23b | N.D. | N.D. | N.D. | MS/RI/ |
| Heptanal | | 158.52±38.33b | N.D. | N.D. | N.D. | MS/RI/ |
| Hexadecane | | 10.9±4.61b | N.D. | N.D. | N.D. | MS/RI/ |
| Hexanoic acid | | 1213.01±183.43b | 13.1±4.03ab | 192.78±28.7ab | 3.5±0.53a | MS/RI/ |
| Mesitylene | | 507.83±129.44b | N.D. | N.D. | N.D. | MS/RI/ |
| Methylpyrazine | | 1361.14±388.39b | 4.73±0.51ab | 514.35±63.82ab | N.D. | MS/RI/ |
| Octadecane | 10.6±3.61b | | N.D. | N.D. | N.D. | MS/RI/ |
| Octanal | 87.96±16.78b | | N.D. | N.D. | N.D. | MS/RI/ |
| Pentanoic acid | 219.14±39.57b | | 1.71±0.04ab | 22.8±1.46ab | 0.52±0.01a | MS/RI/ |
| Propanoic acid | 98.25±15.83b | | N.D. | N.D. | N.D. | MS/RI/ |
| Pyrazine | 13.32±3.37ab | | N.D. | 17.12±3.01b | N.D. | MS/RI/ |
| Pyridine, 2-pentyl- | 100.01±38.66b | | N.D. | N.D. | N.D. | MS/RI/ |
| Pyridine, 3-methoxy- | 76.59±19.2b | | N.D. | N.D. | N.D. | MS/RI/ |
| Pyrrole | 199.44±56.93b | | 2.02±0.65ab | 18.78±2.28ab | N.D. | MS/RI/ |
| Tetradecane | 15.05±6.85b | | N.D. | N.D. | N.D. | MS/RI/ |
| Tridecane | 103.83±25.55a | | 85.77±21.81a | N.D. | N.D. | MS/RI/ |
| Trimethylpyrazine | 267.13±72.02b | | N.D. | 127.11±3.96ab | N.D. | MS/RI/ |
| Octadecane | 10.6±3.61b | | N.D. | N.D. | N.D. | MS/RI/ |
| 1-Octen-3-ol | 1555.27±390.47b | | 19.19±7.5ab | 321.03±40.81ab | 1.19±0.62a | MS/RI/ |
