# Supplementary material for: Formation of characteristic aroma compounds in walnut kernels during thermal processing and their potential recognition by human olfactory receptors
Source: Food Chem X. 2026 Jul 9;37:104142. doi: 10.1016/j.fochx.2026.104142 (PMC13380225; doi:10.1016/j.fochx.2026.104142)
Supplement: Supplementary file 2 — Supplementary material 2 [file mmc2.docx]

**Table 2. Binding Energies between Key Aroma Compounds and Human Olfactory Receptors**

| Receptor | (*E,E*)-2,4-Decadienal | 2-Ethyl-6-methylpyrazine | 1- Hexanol | Ethylpyrazine | (*Z*)-2-Heptenal | Octanal | Pyrazine | (*E*)-2-Decenal | (*E*)-2-Octen-1-ol | (*E,E*)-2,4-Heptadienal | 3-Ethyl-2,5-dimethylpyrazine | 2,6-Diethylpyrazine | 1-Octen-3-ol | 3-Octen-2-one |
| --- | --- | --- | --- | --- | --- | --- | --- | --- | --- | --- | --- | --- | --- | --- |
| OR10A2 | -5.697 | -4.840 | -4.201 | -4.268 | -4.704 | -4.761 | -3.556 | -5.539 | -5.031 | -4.757 | -4.996 | -5.002 | -4.831 | -5.029 |
| OR10A3 | -5.542 | -5.284 | -4.576 | -4.791 | -4.501 | -4.544 | -3.392 | -5.354 | -4.898 | -4.624 | -5.623 | -5.477 | -5.124 | -5.039 |
| OR10A4 | -5.404 | -4.895 | -4.496 | -4.525 | -4.882 | -4.850 | -3.383 | -5.232 | -4.748 | -4.888 | -5.473 | -5.295 | -4.793 | -4.920 |
| OR10A5 | -5.690 | -4.838 | -4.192 | -4.350 | -4.728 | -4.789 | -3.259 | -5.729 | -5.032 | -4.911 | -5.479 | -5.175 | -4.534 | -4.914 |
| OR10A6 | -5.682 | -5.172 | -4.212 | -4.767 | -4.905 | -5.207 | -3.510 | -5.505 | -5.194 | -4.828 | -5.706 | -5.456 | -5.194 | -5.513 |
| OR10A7 | -5.962 | -5.033 | -4.503 | -4.589 | -5.112 | -4.988 | -3.548 | -5.722 | -5.179 | -4.925 | -5.451 | -5.473 | -5.070 | -5.266 |
| OR10AD | -6.039 | -5.204 | -4.732 | -4.843 | -4.946 | -4.967 | -3.399 | -5.621 | -5.427 | -5.057 | -5.816 | -5.469 | -5.163 | -5.456 |
| OR10AG | -5.704 | -5.601 | -4.621 | -4.888 | -4.758 | -4.954 | -3.682 | -5.747 | -5.531 | -5.266 | -5.946 | -5.707 | -5.478 | -5.632 |
| OR10C1 | -5.390 | -5.666 | -4.359 | -4.906 | -4.880 | -4.643 | -3.826 | -5.235 | -5.088 | -4.821 | -6.090 | -5.585 | -5.107 | -5.275 |
| OR10D3 | -4.653 | -4.740 | -3.643 | -4.267 | -4.048 | -3.816 | -3.205 | -4.396 | -4.163 | -3.897 | -4.948 | -4.986 | -4.460 | -4.056 |
| OR10G2 | -5.118 | -5.161 | -3.940 | -4.582 | -4.336 | -4.435 | -3.613 | -4.676 | -4.543 | -4.180 | -5.420 | -5.514 | -4.601 | -4.593 |
| OR10G3 | -5.262 | -5.555 | -4.184 | -4.931 | -4.444 | -4.506 | -3.812 | -5.109 | -4.631 | -4.440 | -5.569 | -5.891 | -5.015 | -4.826 |
| OR10G4 | -5.219 | -5.407 | -4.249 | -4.920 | -4.747 | -4.656 | -3.461 | -4.950 | -4.740 | -4.620 | -5.760 | -5.713 | -4.811 | -4.801 |
| OR10G6 | -5.456 | -5.526 | -4.184 | -4.901 | -4.559 | -4.743 | -3.856 | -5.010 | -4.926 | -4.819 | -5.655 | -5.709 | -4.698 | -5.083 |
| OR10G7 | -4.760 | -4.843 | -3.918 | -4.422 | -4.342 | -4.157 | -3.223 | -4.389 | -4.386 | -4.144 | -5.460 | -5.135 | -4.340 | -4.540 |
| OR10G8 | -5.012 | -5.137 | -3.725 | -4.648 | -4.154 | -4.241 | -3.429 | -4.896 | -4.460 | -4.365 | -5.686 | -5.336 | -4.474 | -4.757 |
| OR10G9 | -4.656 | -5.004 | -3.703 | -4.434 | -4.049 | -4.043 | -3.438 | -4.499 | -4.192 | -4.232 | -5.549 | -5.193 | -4.493 | -4.379 |
| OR10H1 | -6.029 | -5.218 | -4.559 | -4.588 | -5.147 | -5.144 | -3.999 | -5.903 | -5.375 | -5.339 | -4.881 | -5.263 | -5.102 | -5.503 |
| OR10H2 | -5.832 | -5.166 | -4.589 | -4.519 | -5.110 | -5.259 | -4.160 | -5.870 | -5.199 | -5.179 | -5.786 | -5.524 | -5.125 | -5.541 |
| OR10H3 | -5.706 | -5.100 | -4.367 | -4.781 | -4.893 | -5.063 | -4.040 | -5.562 | -5.122 | -4.777 | -5.238 | -5.275 | -4.969 | -5.456 |
| OR10H4 | -6.146 | -5.131 | -4.547 | -4.770 | -5.039 | -5.210 | -4.150 | -5.881 | -5.523 | -5.371 | -5.740 | -5.615 | -5.228 | -5.759 |
| OR10H5 | -6.073 | -5.175 | -4.629 | -5.109 | -5.127 | -5.032 | -4.028 | -5.743 | -5.141 | -5.128 | -5.882 | -5.595 | -5.255 | -5.506 |
| OR10J1 | -6.049 | -5.312 | -4.528 | -5.051 | -5.047 | -5.074 | -3.683 | -5.863 | -5.307 | -5.200 | -5.863 | -5.664 | -5.326 | -5.695 |
| OR10J3 | -5.483 | -5.543 | -4.559 | -4.767 | -4.970 | -4.981 | -3.469 | -5.807 | -5.236 | -4.971 | -5.673 | -5.450 | -5.022 | -5.536 |
| OR10J5 | -6.206 | -5.212 | -4.639 | -4.767 | -5.138 | -5.124 | -3.623 | -5.835 | -5.301 | -5.136 | -5.462 | -5.439 | -5.140 | -5.441 |
| OR10K1 | -5.800 | -5.243 | -4.283 | -4.851 | -4.725 | -4.778 | -3.695 | -5.392 | -5.074 | -5.152 | -5.557 | -5.433 | -5.050 | -5.271 |
| OR10K2 | -5.957 | -5.365 | -4.404 | -4.823 | -4.871 | -4.852 | -3.590 | -5.821 | -5.053 | -5.013 | -5.562 | -5.646 | -5.024 | -5.290 |
| OR10P1 | -5.462 | -5.171 | -4.095 | -4.465 | -4.677 | -4.710 | -3.277 | -5.416 | -4.789 | -4.730 | -5.295 | -5.183 | -4.899 | -5.319 |
| OR10Q1 | -5.937 | -5.385 | -4.516 | -5.039 | -4.874 | -5.257 | -3.662 | -5.785 | -5.254 | -5.240 | -6.209 | -5.798 | -5.364 | -5.699 |
| OR10R2 | -5.950 | -5.018 | -4.178 | -4.319 | -4.674 | -4.955 | -3.521 | -5.571 | -5.134 | -4.947 | -4.928 | -5.287 | -5.121 | -5.292 |
| OR10S1 | -4.647 | -4.570 | -3.590 | -4.132 | -3.931 | -4.094 | -3.128 | -4.308 | -4.136 | -3.966 | -4.784 | -4.783 | -4.224 | -4.349 |
| OR10T2 | -6.093 | -4.824 | -4.426 | -4.263 | -4.983 | -5.065 | -3.240 | -5.875 | -5.231 | -5.249 | -5.332 | -5.121 | -5.239 | -5.411 |
| OR10V1 | -6.251 | -5.042 | -4.338 | -4.649 | -4.774 | -4.963 | -3.438 | -5.940 | -5.110 | -5.060 | -5.535 | -5.352 | -5.103 | -5.345 |
| OR10W1 | -5.321 | -5.282 | -4.551 | -4.816 | -4.861 | -4.901 | -3.629 | -5.637 | -4.817 | -4.611 | -5.552 | -5.497 | -5.263 | -5.227 |
| OR10X1 | -4.923 | -4.976 | -3.876 | -4.500 | -4.191 | -4.145 | -3.427 | -4.697 | -4.516 | -4.339 | -5.365 | -5.270 | -4.367 | -4.777 |
| OR10Z1 | -5.895 | -5.568 | -4.560 | -4.992 | -5.023 | -4.920 | -3.630 | -5.559 | -5.301 | -5.092 | -5.494 | -5.597 | -5.371 | -5.202 |
| OR11A1 | -4.798 | -4.799 | -3.621 | -4.341 | -4.033 | -4.062 | -3.329 | -4.551 | -4.282 | -4.298 | -5.264 | -5.127 | -4.371 | -4.632 |
| OR11G2 | -5.390 | -4.859 | -4.150 | -4.387 | -4.504 | -4.557 | -3.269 | -5.084 | -4.664 | -4.360 | -5.270 | -4.936 | -4.815 | -4.992 |
| OR11H1 | -6.032 | -5.306 | -4.559 | -5.284 | -5.045 | -5.122 | -3.904 | -5.687 | -5.227 | -5.147 | -5.694 | -5.718 | -5.470 | -5.535 |
| OR11H2 | -5.856 | -5.791 | -4.981 | -5.639 | -5.429 | -5.463 | -3.901 | -5.570 | -5.366 | -5.307 | -6.050 | -6.144 | -5.888 | -5.981 |
| OR11H4 | -5.242 | -5.593 | -5.062 | -5.400 | -5.622 | -5.710 | -4.064 | -5.505 | -5.843 | -5.131 | -5.517 | -5.682 | -5.775 | -5.817 |
| OR11H6 | -5.822 | -5.508 | -4.744 | -5.318 | -4.979 | -5.230 | -3.729 | -5.770 | -5.284 | -5.145 | -5.756 | -5.790 | -5.700 | -5.654 |
| OR11HC | -4.800 | -5.647 | -4.541 | -5.447 | -5.021 | -5.183 | -4.035 | -4.217 | -5.248 | -4.609 | -5.374 | -5.781 | -5.629 | -5.547 |
| OR11L1 | -5.454 | -5.552 | -4.738 | -4.767 | -5.323 | -5.604 | -3.435 | -5.724 | -5.353 | -4.879 | -6.026 | -5.646 | -5.282 | -5.399 |
| OR12D2 | -5.290 | -5.450 | -4.270 | -4.960 | -4.771 | -4.911 | -3.692 | -5.278 | -4.938 | -4.542 | -6.166 | -5.749 | -4.938 | -5.191 |
| OR12D3 | -5.050 | -5.581 | -4.554 | -5.220 | -5.015 | -4.911 | -3.912 | -5.121 | -4.949 | -5.112 | -5.987 | -5.708 | -5.197 | -5.253 |
| OR13A1 | -5.518 | -5.611 | -4.800 | -4.896 | -5.469 | -5.533 | -3.426 | -5.835 | -5.331 | -4.916 | -6.104 | -5.672 | -5.356 | -5.569 |
| OR13C2 | -4.749 | -4.680 | -3.691 | -4.343 | -4.121 | -4.148 | -3.389 | -4.223 | -4.136 | -4.226 | -4.817 | -4.921 | -4.210 | -4.322 |
| OR13C3 | -5.397 | -5.042 | -3.932 | -4.532 | -4.396 | -4.392 | -3.522 | -5.088 | -4.523 | -4.583 | -5.240 | -5.080 | -4.561 | -4.674 |
| OR13C4 | -5.656 | -4.975 | -4.040 | -4.691 | -4.606 | -4.532 | -3.533 | -5.019 | -4.866 | -4.783 | -5.535 | -5.276 | -4.458 | -4.984 |
| OR13C5 | -4.731 | -5.072 | -4.050 | -4.690 | -4.467 | -4.565 | -3.566 | -4.666 | -4.412 | -4.401 | -5.578 | -5.320 | -4.400 | -4.537 |
| OR13C8 | -5.328 | -4.933 | -4.161 | -4.505 | -4.427 | -4.700 | -3.435 | -5.034 | -4.712 | -4.750 | -5.179 | -5.321 | -4.805 | -4.923 |
| OR13C9 | -4.997 | -4.704 | -3.738 | -4.123 | -4.058 | -4.005 | -3.445 | -4.740 | -4.288 | -4.245 | -4.927 | -4.908 | -4.212 | -4.482 |
| OR13D1 | -4.934 | -4.841 | -4.191 | -4.330 | -4.255 | -4.594 | -3.305 | -4.480 | -4.795 | -4.737 | -5.216 | -5.129 | -4.600 | -4.761 |
| OR13F1 | -4.845 | -5.050 | -3.868 | -4.557 | -4.189 | -4.302 | -3.678 | -4.548 | -4.297 | -4.259 | -5.434 | -5.141 | -4.366 | -4.664 |
| OR13G1 | -4.817 | -4.772 | -3.836 | -4.041 | -4.122 | -4.360 | -3.247 | -4.494 | -4.506 | -4.232 | -4.571 | -4.680 | -4.276 | -4.478 |
| OR13H1 | -4.778 | -4.722 | -3.898 | -4.518 | -4.337 | -4.317 | -3.378 | -4.645 | -4.388 | -4.548 | -5.369 | -4.946 | -4.565 | -4.641 |
| OR13J1 | -4.875 | -5.095 | -4.316 | -4.830 | -4.717 | -4.314 | -3.686 | -4.656 | -4.573 | -4.518 | -5.555 | -5.228 | -4.251 | -4.641 |
| OR14A2 | -5.829 | -4.958 | -4.353 | -4.539 | -4.927 | -4.971 | -3.487 | -5.686 | -5.121 | -5.037 | -5.217 | -5.126 | -5.017 | -5.244 |
| OR14AG | -5.651 | -5.284 | -4.407 | -4.567 | -4.594 | -4.812 | -3.684 | -5.202 | -4.896 | -4.888 | -5.657 | -5.613 | -4.775 | -4.998 |
| OR14CZ | -5.337 | -4.560 | -4.084 | -4.186 | -4.401 | -4.599 | -3.292 | -5.183 | -4.610 | -4.316 | -4.924 | -4.997 | -4.691 | -4.812 |
| OR14I1 | -4.874 | -4.392 | -3.921 | -4.529 | -4.246 | -4.103 | -3.511 | -4.404 | -4.412 | -4.444 | -4.582 | -4.679 | -4.393 | -4.728 |
| OR14J1 | -4.983 | -4.358 | -3.965 | -3.912 | -4.219 | -4.155 | -3.113 | -4.482 | -4.455 | -4.572 | -4.895 | -4.606 | -4.458 | -4.530 |
| OR14K1 | -5.127 | -4.729 | -3.947 | -4.279 | -4.133 | -4.057 | -3.086 | -4.679 | -4.577 | -4.578 | -4.901 | -4.997 | -4.418 | -4.801 |
| OR2A12 | -5.067 | -4.795 | -4.519 | -4.657 | -5.079 | -5.301 | -3.509 | -5.150 | -5.578 | -5.255 | -4.618 | -4.773 | -5.228 | -5.726 |
| OR2A14 | -5.898 | -4.941 | -4.263 | -4.299 | -4.686 | -4.774 | -3.202 | -5.611 | -5.033 | -4.913 | -4.978 | -5.213 | -4.905 | -5.107 |
| OR2A25 | -4.641 | -4.206 | -3.670 | -3.949 | -4.124 | -4.439 | -2.983 | -4.479 | -4.253 | -3.933 | -4.663 | -4.439 | -4.288 | -4.439 |
| OR2A42 | -5.503 | -5.204 | -4.101 | -4.589 | -4.648 | -4.724 | -3.277 | -5.321 | -4.871 | -4.826 | -5.523 | -5.421 | -4.694 | -5.130 |
| OR2AE1 | -5.069 | -4.664 | -3.859 | -4.191 | -4.458 | -4.569 | -3.258 | -4.848 | -4.516 | -4.554 | -4.772 | -4.810 | -4.211 | -4.901 |
| OR2AG1 | -5.828 | -5.374 | -4.612 | -4.745 | -5.140 | -4.909 | -3.544 | -5.511 | -5.206 | -5.039 | -5.559 | -5.678 | -5.178 | -5.347 |
| OR2AG2 | -6.193 | -5.476 | -4.625 | -4.761 | -5.219 | -5.178 | -3.749 | -5.610 | -5.313 | -5.521 | -6.182 | -5.879 | -5.329 | -5.797 |
| OR2AJ1 | -5.573 | -5.313 | -4.334 | -4.909 | -4.769 | -4.937 | -3.692 | -5.398 | -4.921 | -4.636 | -5.392 | -5.745 | -5.209 | -5.148 |
| OR2AK2 | -6.061 | -5.650 | -5.089 | -5.283 | -5.494 | -5.230 | -4.250 | -5.772 | -5.756 | -5.417 | -5.324 | -5.672 | -5.378 | -5.922 |
| OR2AP1 | -4.981 | -4.268 | -3.849 | -3.918 | -4.028 | -3.860 | -2.907 | -4.891 | -4.354 | -4.281 | -4.956 | -4.663 | -4.338 | -4.526 |
| OR2AT4 | -4.781 | -4.509 | -3.776 | -4.103 | -4.210 | -4.259 | -3.224 | -4.602 | -4.386 | -4.251 | -4.861 | -4.803 | -4.325 | -4.485 |
| OR2T10 | -5.943 | -5.692 | -4.676 | -5.259 | -5.102 | -5.243 | -3.591 | -5.679 | -5.671 | -5.295 | -5.928 | -5.860 | -5.207 | -5.509 |
| OR2T11 | -4.699 | -4.479 | -4.175 | -3.996 | -4.162 | -4.608 | -2.914 | -4.654 | -4.670 | -4.389 | -4.805 | -4.432 | -4.680 | -4.892 |
| OR2T12 | -5.051 | -4.702 | -4.082 | -4.352 | -4.708 | -4.593 | -3.109 | -5.058 | -4.662 | -4.510 | -5.176 | -4.920 | -4.857 | -4.821 |
| OR2T27 | -6.006 | -4.555 | -4.469 | -4.436 | -4.762 | -5.095 | -3.133 | -5.865 | -4.929 | -4.956 | -4.189 | -4.312 | -5.252 | -5.318 |
| OR2T29 | -5.100 | -4.384 | -4.461 | -4.005 | -3.838 | -4.048 | -3.179 | -4.822 | -4.357 | -3.971 | -4.435 | -4.625 | -4.326 | -4.290 |
| OR2T33 | -5.580 | -5.151 | -4.476 | -4.590 | -4.905 | -4.872 | -3.247 | -5.396 | -5.133 | -4.688 | -5.590 | -5.440 | -4.673 | -5.142 |
| OR2T34 | -5.335 | -4.391 | -3.998 | -4.216 | -4.537 | -4.806 | -3.119 | -5.203 | -4.718 | -4.541 | -4.598 | -4.337 | -4.757 | -4.954 |
| OR2T35 | -5.578 | -5.252 | -4.624 | -4.732 | -5.089 | -4.847 | -3.217 | -5.373 | -5.137 | -5.145 | -5.753 | -5.685 | -5.173 | -5.401 |
| OR4A15 | -5.799 | -5.110 | -4.653 | -4.666 | -5.071 | -5.102 | -3.308 | -5.712 | -5.384 | -5.268 | -4.882 | -5.395 | -5.245 | -5.418 |
| OR4A16 | -5.232 | -5.040 | -4.185 | -4.486 | -4.638 | -4.625 | -3.367 | -5.156 | -4.981 | -4.705 | -5.591 | -5.425 | -4.932 | -5.000 |
| OR4A47 | -5.483 | -5.075 | -4.089 | -4.668 | -4.427 | -4.425 | -3.670 | -5.307 | -4.885 | -4.553 | -5.566 | -5.364 | -4.970 | -5.013 |
| OR4C45 | -5.046 | -3.772 | -3.824 | -3.895 | -4.093 | -4.244 | -3.570 | -4.800 | -4.432 | -4.275 | -3.721 | -4.121 | -4.548 | -4.527 |
| OR4C46 | -5.233 | -4.864 | -4.044 | -4.468 | -4.310 | -4.445 | -3.504 | -5.080 | -4.628 | -4.632 | -5.208 | -5.219 | -4.564 | -4.859 |
| OR4F15 | -5.514 | -4.924 | -4.157 | -4.439 | -4.811 | -4.757 | -3.374 | -5.234 | -5.022 | -4.984 | -5.569 | -5.441 | -4.996 | -5.211 |
| OR4F16 | -5.202 | -4.724 | -4.135 | -4.118 | -4.499 | -4.450 | -3.189 | -5.066 | -4.780 | -4.426 | -5.054 | -5.005 | -4.752 | -4.881 |
| OR4F17 | -4.567 | -4.474 | -3.844 | -4.073 | -4.320 | -4.280 | -3.167 | -4.589 | -4.573 | -4.421 | -4.412 | -4.655 | -4.476 | -4.858 |
| OR4F21 | -5.279 | -4.967 | -4.142 | -4.462 | -4.642 | -4.805 | -3.219 | -5.341 | -4.749 | -4.988 | -5.173 | -5.299 | -5.092 | -5.216 |
| OR4F29 | -5.198 | -4.672 | -4.078 | -4.144 | -4.496 | -4.536 | -3.182 | -5.263 | -4.720 | -4.437 | -5.059 | -5.036 | -4.729 | -4.863 |
| OR51A2 | -4.073 | -4.289 | -3.480 | -3.762 | -3.951 | -3.900 | -2.788 | -4.064 | -3.875 | -3.984 | -4.401 | -4.489 | -3.831 | -4.040 |
| OR51A4 | -4.430 | -4.484 | -3.692 | -4.224 | -4.112 | -4.139 | -3.077 | -4.449 | -3.987 | -3.997 | -4.767 | -4.596 | -4.409 | -4.411 |
| OR51A7 | -4.812 | -4.414 | -3.788 | -4.190 | -4.390 | -4.467 | -3.100 | -4.831 | -4.544 | -4.444 | -4.663 | -4.730 | -4.397 | -4.730 |
| OR51B2 | -5.402 | -4.637 | -4.092 | -4.170 | -4.681 | -4.742 | -2.956 | -5.004 | -4.607 | -4.731 | -4.901 | -5.039 | -4.550 | -5.048 |
| OR51B4 | -4.542 | -4.312 | -3.627 | -3.777 | -4.024 | -3.850 | -2.992 | -4.324 | -4.207 | -4.085 | -4.883 | -4.645 | -4.110 | -4.370 |
| OR51B5 | -4.984 | -4.522 | -3.911 | -4.065 | -4.326 | -4.117 | -3.085 | -4.667 | -4.395 | -4.148 | -4.698 | -4.619 | -4.298 | -4.704 |
| OR51B6 | -4.909 | -4.663 | -3.791 | -4.212 | -4.162 | -4.354 | -3.186 | -4.804 | -4.323 | -4.266 | -4.876 | -4.937 | -4.496 | -4.524 |
| OR51D1 | -3.676 | -4.050 | -3.816 | -3.783 | -3.955 | -3.716 | -3.199 | -3.877 | -4.097 | -3.487 | -4.427 | -4.329 | -3.915 | -4.110 |
| OR51E1 | -5.327 | -4.521 | -3.910 | -4.299 | -4.362 | -4.326 | -3.192 | -4.765 | -4.602 | -4.475 | -5.068 | -4.854 | -4.312 | -4.601 |
| OR51E2 | -4.780 | -4.637 | -4.085 | -4.264 | -4.561 | -4.471 | -2.959 | -4.729 | -4.544 | -4.661 | -4.624 | -4.677 | -4.499 | -4.644 |
| OR51F1 | -5.032 | -4.832 | -3.626 | -4.443 | -4.031 | -4.247 | -3.416 | -4.830 | -4.460 | -4.310 | -5.195 | -5.103 | -4.363 | -4.660 |
| OR51F2 | -4.926 | -4.812 | -3.677 | -4.377 | -4.127 | -4.083 | -3.084 | -4.692 | -4.362 | -4.379 | -5.082 | -5.029 | -4.541 | -4.725 |
| OR51G1 | -4.658 | -4.312 | -3.684 | -4.119 | -4.262 | -4.248 | -3.261 | -4.464 | -4.386 | -4.508 | -4.371 | -4.480 | -4.245 | -4.430 |
| OR51G2 | -4.696 | -4.554 | -3.705 | -3.982 | -4.136 | -4.052 | -2.874 | -4.404 | -4.161 | -4.114 | -4.796 | -4.791 | -3.975 | -4.467 |
| OR51I1 | -4.961 | -4.611 | -3.880 | -4.080 | -4.186 | -4.116 | -3.044 | -4.621 | -4.540 | -4.442 | -4.895 | -4.894 | -4.266 | -4.533 |
| OR51I2 | -4.644 | -4.600 | -3.638 | -4.124 | -4.017 | -3.919 | -3.035 | -4.367 | -4.303 | -4.106 | -4.897 | -4.893 | -4.268 | -4.355 |
| OR51J1 | -4.826 | -4.148 | -3.953 | -4.238 | -4.169 | -4.300 | -3.397 | -4.975 | -4.373 | -4.193 | -4.199 | -4.204 | -4.123 | -4.633 |
| OR51L1 | -4.762 | -4.239 | -3.967 | -4.253 | -4.311 | -4.229 | -3.374 | -4.373 | -4.243 | -4.437 | -4.611 | -4.560 | -4.596 | -4.707 |
| OR51M1 | -4.345 | -4.488 | -3.384 | -3.977 | -3.852 | -3.732 | -2.966 | -4.205 | -3.835 | -3.722 | -4.856 | -4.671 | -3.806 | -4.059 |
| OR51Q1 | -4.744 | -4.952 | -3.901 | -4.723 | -4.737 | -4.224 | -3.386 | -4.721 | -4.346 | -4.104 | -5.272 | -5.062 | -4.749 | -4.648 |
| OR51S1 | -4.338 | -4.184 | -3.448 | -3.767 | -3.623 | -3.549 | -3.000 | -4.231 | -3.973 | -3.848 | -4.464 | -4.430 | -3.954 | -4.146 |
| OR51T1 | -4.901 | -4.769 | -4.122 | -4.261 | -4.282 | -4.429 | -3.506 | -4.915 | -4.643 | -4.354 | -4.843 | -4.940 | -4.368 | -4.707 |
| OR51V1 | -5.083 | -4.636 | -4.184 | -4.523 | -4.676 | -4.440 | -3.667 | -4.886 | -4.651 | -4.317 | -4.874 | -4.865 | -4.807 | -4.786 |
| OR52A1 | -5.121 | -4.845 | -3.837 | -4.132 | -4.156 | -4.256 | -3.247 | -4.943 | -4.329 | -4.310 | -5.125 | -4.557 | -4.527 | -4.614 |
| OR52A4 | -5.218 | -4.749 | -4.059 | -4.441 | -4.499 | -4.693 | -3.363 | -5.135 | -4.795 | -4.707 | -5.209 | -4.971 | -4.594 | -5.123 |
| OR52A5 | -5.098 | -4.895 | -4.102 | -4.200 | -4.538 | -4.738 | -3.177 | -4.860 | -4.649 | -4.681 | -4.909 | -5.305 | -4.774 | -5.128 |
| OR52B2 | -5.438 | -4.634 | -3.978 | -4.034 | -4.177 | -4.606 | -2.923 | -5.126 | -4.775 | -4.583 | -5.281 | -4.977 | -4.618 | -4.917 |
| OR52B4 | -5.332 | -5.167 | -4.194 | -4.493 | -4.595 | -4.748 | -3.301 | -5.246 | -4.968 | -4.900 | -5.258 | -5.469 | -4.881 | -5.172 |
| OR52B6 | -4.687 | -4.321 | -3.547 | -3.920 | -3.966 | -3.924 | -2.879 | -4.486 | -4.117 | -4.020 | -4.798 | -4.580 | -4.103 | -4.171 |
| OR52D1 | -5.164 | -4.800 | -3.845 | -4.165 | -4.349 | -4.558 | -3.179 | -4.979 | -4.718 | -4.452 | -5.230 | -4.825 | -4.672 | -4.896 |
| OR52E2 | -5.064 | -4.165 | -3.530 | -3.929 | -4.093 | -4.201 | -2.894 | -4.841 | -4.319 | -4.234 | -4.674 | -4.485 | -4.194 | -4.568 |
| OR52E4 | -4.849 | -4.384 | -4.036 | -3.844 | -3.757 | -3.826 | -2.720 | -4.140 | -4.526 | -4.344 | -4.768 | -4.454 | -4.145 | -4.421 |
| OR52E5 | -5.480 | -4.746 | -4.071 | -4.290 | -4.496 | -4.788 | -3.451 | -5.277 | -4.921 | -4.838 | -5.282 | -5.062 | -4.610 | -4.969 |
| OR52E6 | -5.620 | -4.576 | -4.362 | -4.037 | -5.046 | -5.119 | -3.064 | -5.503 | -5.239 | -5.022 | -5.126 | -4.877 | -4.916 | -5.462 |
| OR52E8 | -4.827 | -4.658 | -3.782 | -4.248 | -4.097 | -4.254 | -3.135 | -4.479 | -4.376 | -4.216 | -4.962 | -4.841 | -4.380 | -4.363 |
| OR52H1 | -5.145 | -4.684 | -3.939 | -4.263 | -4.346 | -4.314 | -3.187 | -4.890 | -4.625 | -4.610 | -5.121 | -4.924 | -4.560 | -4.673 |
| OR52I1 | -4.627 | -4.289 | -3.432 | -3.883 | -3.784 | -4.054 | -3.087 | -4.425 | -4.165 | -4.254 | -4.569 | -4.602 | -3.877 | -4.163 |
| OR52I2 | -4.390 | -4.605 | -3.551 | -4.223 | -3.864 | -3.700 | -3.273 | -4.151 | -4.033 | -4.185 | -4.790 | -4.742 | -4.324 | -4.150 |
| OR52J3 | -5.978 | -5.013 | -4.456 | -4.558 | -4.621 | -5.073 | -3.606 | -5.829 | -5.134 | -5.005 | -5.437 | -5.297 | -4.982 | -5.444 |
| OR52K1 | -4.932 | -4.279 | -3.784 | -4.004 | -4.083 | -4.252 | -3.049 | -4.820 | -4.530 | -4.280 | -4.417 | -4.373 | -4.470 | -4.639 |
| OR52K2 | -5.078 | -4.372 | -3.832 | -4.132 | -4.328 | -4.363 | -3.105 | -5.048 | -4.485 | -4.355 | -4.964 | -4.692 | -4.343 | -4.645 |
| OR52L1 | -5.446 | -4.952 | -3.969 | -4.358 | -4.546 | -4.417 | -3.365 | -5.356 | -4.712 | -4.632 | -5.138 | -5.207 | -4.935 | -4.837 |
| OR52M1 | -5.234 | -4.333 | -3.948 | -4.279 | -4.341 | -4.321 | -3.300 | -4.885 | -4.473 | -4.412 | -4.833 | -4.595 | -4.607 | -4.755 |
| OR52N1 | -4.857 | -4.768 | -3.702 | -4.041 | -4.169 | -4.252 | -3.243 | -4.700 | -4.470 | -4.428 | -5.105 | -4.796 | -4.261 | -4.745 |
| OR52N2 | -5.140 | -4.378 | -3.759 | -4.240 | -4.148 | -4.196 | -2.975 | -4.900 | -4.695 | -4.359 | -4.792 | -4.706 | -4.492 | -4.794 |
| OR52N4 | -5.076 | -4.815 | -4.263 | -4.555 | -4.581 | -4.541 | -3.412 | -4.973 | -4.511 | -4.421 | -5.229 | -5.026 | -4.471 | -4.778 |
| OR52N5 | -4.988 | -4.653 | -4.047 | -4.193 | -4.431 | -4.577 | -3.146 | -5.127 | -4.575 | -4.348 | -5.027 | -4.888 | -4.451 | -4.814 |
| OR52R1 | -4.931 | -4.832 | -3.886 | -4.537 | -4.074 | -4.431 | -3.309 | -4.951 | -4.424 | -4.431 | -5.085 | -4.982 | -4.709 | -4.694 |
| OR52W1 | -4.919 | -5.010 | -3.722 | -4.407 | -4.192 | -4.063 | -3.398 | -4.828 | -4.067 | -4.161 | -5.360 | -5.191 | -4.765 | -4.625 |
| OR56A1 | -5.011 | -4.409 | -3.677 | -3.827 | -4.430 | -3.939 | -2.960 | -4.612 | -4.486 | -4.923 | -4.561 | -4.521 | -4.316 | -4.611 |
| OR56A3 | -4.664 | -4.185 | -3.936 | -3.873 | -4.083 | -4.107 | -3.031 | -4.264 | -4.415 | -3.945 | -4.453 | -4.433 | -3.932 | -4.284 |
| OR56A4 | -4.472 | -4.706 | -3.623 | -4.513 | -4.020 | -3.601 | -3.690 | -4.421 | -3.994 | -3.934 | -4.769 | -4.870 | -3.939 | -4.134 |
| OR56A5 | -4.127 | -4.728 | -3.368 | -4.260 | -3.802 | -3.474 | -3.325 | -3.818 | -3.878 | -3.685 | -4.930 | -4.920 | -4.173 | -4.043 |
| OR56B1 | -4.952 | -5.018 | -4.188 | -4.687 | -4.575 | -4.366 | -3.473 | -4.707 | -4.545 | -4.455 | -5.508 | -5.137 | -4.612 | -4.753 |
| OR56B4 | -4.761 | -4.385 | -3.733 | -3.859 | -4.017 | -3.962 | -2.913 | -4.458 | -4.109 | -4.032 | -4.517 | -4.667 | -4.117 | -4.277 |
| OR5AC2 | -4.834 | -4.643 | -4.079 | -4.222 | -4.137 | -4.385 | -3.216 | -4.909 | -4.695 | -4.222 | -4.917 | -4.789 | -4.377 | -4.629 |
| OR5AK2 | -5.030 | -5.133 | -3.991 | -4.616 | -4.600 | -4.660 | -3.503 | -5.064 | -4.711 | -4.591 | -5.526 | -5.474 | -4.745 | -5.094 |
| OR5AN1 | -5.111 | -4.638 | -3.700 | -4.194 | -4.089 | -4.267 | -3.146 | -4.783 | -4.281 | -4.555 | -4.769 | -4.781 | -4.144 | -4.423 |
| OR5AP2 | -4.997 | -4.947 | -4.139 | -4.480 | -4.393 | -4.510 | -3.456 | -4.769 | -4.575 | -4.390 | -5.187 | -5.265 | -4.634 | -4.716 |
| OR5AR1 | -5.255 | -4.681 | -4.144 | -4.282 | -4.186 | -4.323 | -3.016 | -4.955 | -4.578 | -4.250 | -4.882 | -4.962 | -4.519 | -4.657 |
| OR5AS1 | -4.812 | -4.395 | -3.860 | -3.965 | -4.276 | -4.150 | -2.843 | -4.576 | -4.751 | -4.411 | -4.940 | -4.693 | -4.227 | -4.598 |
| OR5AU1 | -5.249 | -4.724 | -3.855 | -4.618 | -4.290 | -4.232 | -3.482 | -4.898 | -4.465 | -4.299 | -5.108 | -4.854 | -4.408 | -4.788 |
| OR5B21 | -5.206 | -4.636 | -4.079 | -4.222 | -4.521 | -4.731 | -3.174 | -5.121 | -4.673 | -4.527 | -5.110 | -4.992 | -4.628 | -5.019 |
| OR5H14 | -5.511 | -4.505 | -4.555 | -4.251 | -4.940 | -4.764 | -3.307 | -5.262 | -5.235 | -5.052 | -4.597 | -4.768 | -4.916 | -5.142 |
| OR5H15 | -4.875 | -4.251 | -3.788 | -4.068 | -4.173 | -4.133 | -3.101 | -4.747 | -4.292 | -4.197 | -4.505 | -4.541 | -4.290 | -4.483 |
| OR6C65 | -4.858 | -4.658 | -3.854 | -4.224 | -4.129 | -4.096 | -3.170 | -4.893 | -4.479 | -4.284 | -4.796 | -4.984 | -4.372 | -4.658 |
| OR6C68 | -5.554 | -4.332 | -4.082 | -3.970 | -4.508 | -4.673 | -2.951 | -5.382 | -4.795 | -4.735 | -4.640 | -4.559 | -4.924 | -5.044 |
| OR6C70 | -4.757 | -4.159 | -3.636 | -3.759 | -3.932 | -3.992 | -3.166 | -4.723 | -4.332 | -4.024 | -4.581 | -4.494 | -4.442 | -4.630 |
| OR6C74 | -4.730 | -4.636 | -3.979 | -4.169 | -4.162 | -4.202 | -3.211 | -4.685 | -4.450 | -3.983 | -5.000 | -4.832 | -4.493 | -4.305 |
| OR6C75 | -5.117 | -4.392 | -4.049 | -4.044 | -4.234 | -4.437 | -2.868 | -4.804 | -4.835 | -4.503 | -4.546 | -4.677 | -4.351 | -5.028 |
| OR6C76 | -4.907 | -4.445 | -4.167 | -4.067 | -4.205 | -4.110 | -3.097 | -4.631 | -4.687 | -4.247 | -4.669 | -4.675 | -4.279 | -4.598 |
| OR7E24 | -5.019 | -5.490 | -4.369 | -4.737 | -4.502 | -4.664 | -3.738 | -5.378 | -4.974 | -4.464 | -5.761 | -5.771 | -5.140 | -5.152 |
| OR1A1 | -6.249 | -5.285 | -4.633 | -4.847 | -5.133 | -5.191 | -3.531 | -5.563 | -5.443 | -5.399 | -5.502 | -5.592 | -5.241 | -5.585 |
| OR1A2 | -5.834 | -5.299 | -4.492 | -4.558 | -5.009 | -5.192 | -3.125 | -5.705 | -5.158 | -5.351 | -5.595 | -5.449 | -5.036 | -5.421 |
| OR1B1 | -5.745 | -4.872 | -4.182 | -4.491 | -4.559 | -4.831 | -3.240 | -5.370 | -4.986 | -4.987 | -5.341 | -5.250 | -4.745 | -5.247 |
| OR1C1 | -4.721 | -4.360 | -3.684 | -3.768 | -3.956 | -4.134 | -2.983 | -4.715 | -4.349 | -4.194 | -4.824 | -4.669 | -4.472 | -4.516 |
| OR1D2 | -6.020 | -5.079 | -4.290 | -4.572 | -4.929 | -5.272 | -3.303 | -5.779 | -5.122 | -5.152 | -5.395 | -5.545 | -5.304 | -5.632 |
| OR1D4 | -6.236 | -5.154 | -4.466 | -4.465 | -5.111 | -5.263 | -3.332 | -5.910 | -5.297 | -5.212 | -5.702 | -5.572 | -5.319 | -5.481 |
| OR1D5 | -6.251 | -4.936 | -4.614 | -4.559 | -5.108 | -4.915 | -3.093 | -6.042 | -5.313 | -5.388 | -5.472 | -5.462 | -5.384 | -5.599 |
| OR1E1 | -4.591 | -4.215 | -3.617 | -3.798 | -4.017 | -3.970 | -3.029 | -4.400 | -4.075 | -4.266 | -4.582 | -4.549 | -4.158 | -4.310 |
| OR1E2 | -4.438 | -4.423 | -3.760 | -3.960 | -4.144 | -4.063 | -3.040 | -4.456 | -4.058 | -4.197 | -4.606 | -4.711 | -4.464 | -4.564 |
| OR1F1 | -5.411 | -5.228 | -4.401 | -4.909 | -4.628 | -4.711 | -3.278 | -5.154 | -4.858 | -4.686 | -5.432 | -5.627 | -5.106 | -5.168 |
| OR1FC | -5.551 | -4.478 | -4.374 | -4.548 | -4.583 | -4.908 | -3.329 | -5.288 | -4.971 | -5.128 | -4.943 | -4.829 | -4.785 | -5.337 |
| OR1G1 | -4.234 | -3.881 | -3.495 | -3.654 | -3.813 | -3.692 | -2.787 | -4.233 | -3.853 | -3.675 | -4.389 | -4.141 | -3.783 | -4.072 |
| OR1I1 | -4.052 | -4.217 | -3.454 | -3.737 | -3.386 | -3.455 | -2.823 | -3.934 | -3.907 | -3.720 | -4.556 | -4.414 | -4.135 | -3.910 |
| OR1J1 | -5.308 | -4.689 | -4.212 | -4.161 | -4.578 | -4.702 | -3.159 | -5.084 | -5.011 | -5.002 | -4.914 | -4.896 | -4.527 | -5.193 |
| OR1J2 | -4.742 | -4.092 | -3.760 | -3.799 | -4.089 | -4.106 | -2.833 | -4.470 | -4.403 | -4.032 | -4.542 | -4.487 | -4.149 | -4.293 |
| OR1J4 | -4.462 | -4.241 | -3.661 | -3.770 | -4.006 | -3.976 | -2.865 | -4.487 | -4.101 | -4.067 | -4.546 | -4.436 | -4.150 | -4.391 |
| OR1K1 | -5.552 | -4.861 | -4.140 | -4.695 | -4.601 | -4.725 | -3.456 | -5.172 | -5.011 | -4.670 | -5.013 | -5.018 | -4.768 | -5.175 |
| OR1L1 | -5.341 | -4.787 | -4.207 | -4.599 | -4.533 | -4.538 | -3.512 | -4.978 | -4.921 | -4.786 | -5.006 | -5.067 | -4.567 | -4.970 |
| OR1L3 | -5.256 | -4.908 | -4.257 | -4.666 | -4.418 | -4.411 | -3.626 | -5.160 | -4.823 | -4.563 | -5.453 | -5.149 | -4.776 | -4.760 |
| OR1L4 | -6.402 | -5.030 | -4.608 | -4.519 | -4.926 | -5.049 | -3.413 | -6.054 | -5.176 | -5.243 | -5.279 | -5.335 | -5.121 | -5.245 |
| OR1L6 | -5.493 | -5.030 | -4.313 | -4.485 | -4.557 | -4.705 | -3.091 | -5.305 | -4.921 | -4.643 | -5.110 | -5.202 | -4.670 | -5.023 |
| OR1L8 | -5.385 | -5.477 | -4.106 | -4.743 | -4.799 | -4.670 | -3.473 | -5.197 | -4.945 | -4.801 | -5.652 | -5.473 | -5.173 | -5.137 |
| OR1M1 | -5.712 | -4.755 | -4.185 | -4.379 | -4.689 | -4.753 | -3.295 | -5.286 | -5.009 | -4.940 | -5.313 | -5.130 | -4.702 | -5.083 |
| OR1N1 | -5.680 | -5.149 | -4.548 | -4.599 | -4.837 | -4.952 | -3.100 | -5.529 | -5.314 | -4.975 | -5.817 | -5.534 | -5.151 | -5.263 |
| OR1N2 | -5.566 | -5.157 | -4.485 | -4.656 | -4.709 | -4.893 | -3.318 | -5.376 | -5.042 | -4.924 | -5.698 | -5.372 | -5.261 | -5.433 |
| OR1Q1 | -5.431 | -4.789 | -3.984 | -4.210 | -4.318 | -4.553 | -3.229 | -5.118 | -4.758 | -4.607 | -5.418 | -5.119 | -4.798 | -4.974 |
| OR1S1 | -4.920 | -4.350 | -3.605 | -3.909 | -4.179 | -4.278 | -2.980 | -4.461 | -4.380 | -4.323 | -4.647 | -4.689 | -4.323 | -4.556 |
| OR1S2 | -5.002 | -4.218 | -3.549 | -3.951 | -4.147 | -4.163 | -3.006 | -4.654 | -4.506 | -4.304 | -4.562 | -4.566 | -4.317 | -4.462 |
| OR2A1 | -5.519 | -5.211 | -4.079 | -4.586 | -4.645 | -4.651 | -3.274 | -4.963 | -4.767 | -4.846 | -5.521 | -5.420 | -4.634 | -4.973 |
| OR2A2 | -5.363 | -4.861 | -4.171 | -4.261 | -4.689 | -4.800 | -3.204 | -5.477 | -4.953 | -4.819 | -4.853 | -5.329 | -4.913 | -5.215 |
| OR2A4 | -5.199 | -4.792 | -4.090 | -4.156 | -4.493 | -4.547 | -3.155 | -4.846 | -4.610 | -4.460 | -5.205 | -4.983 | -4.846 | -4.785 |
| OR2A5 | -5.960 | -5.039 | -4.339 | -4.753 | -5.057 | -5.236 | -3.456 | -5.662 | -5.148 | -5.141 | -5.147 | -5.333 | -5.091 | -5.506 |
| OR2A7 | -4.781 | -4.572 | -3.638 | -4.067 | -3.794 | -4.012 | -3.240 | -4.421 | -4.302 | -4.107 | -4.823 | -4.867 | -4.232 | -4.328 |
| OR2B2 | -6.003 | -5.279 | -4.672 | -4.956 | -5.167 | -5.201 | -4.107 | -5.851 | -5.392 | -5.368 | -5.676 | -5.652 | -5.337 | -5.541 |
| OR2B3 | -4.626 | -4.608 | -4.374 | -4.640 | -5.032 | -4.970 | -3.923 | -4.804 | -5.104 | -4.550 | -4.659 | -4.626 | -5.024 | -4.324 |
| OR2B6 | -5.216 | -5.011 | -4.137 | -4.448 | -4.388 | -4.431 | -3.479 | -5.237 | -4.752 | -4.474 | -5.102 | -5.443 | -4.778 | -5.063 |
| OR2BB | -5.045 | -5.352 | -4.440 | -4.862 | -5.013 | -5.278 | -3.618 | -5.438 | -4.863 | -4.517 | -5.488 | -5.623 | -5.105 | -5.250 |
| OR2C1 | -5.959 | -5.210 | -4.515 | -4.706 | -4.957 | -4.877 | -3.628 | -5.535 | -5.304 | -5.023 | -5.918 | -5.659 | -5.343 | -5.333 |
| OR2C3 | -5.333 | -4.546 | -3.813 | -4.286 | -4.443 | -4.561 | -3.338 | -4.949 | -4.708 | -4.575 | -4.944 | -4.816 | -4.601 | -4.811 |
| OR2D2 | -5.852 | -5.134 | -4.403 | -4.642 | -5.167 | -4.722 | -3.222 | -5.319 | -4.958 | -5.179 | -5.454 | -5.454 | -4.882 | -5.381 |
| OR2D3 | -6.059 | -5.466 | -4.504 | -4.630 | -4.775 | -4.905 | -3.325 | -5.806 | -5.259 | -4.918 | -5.780 | -5.639 | -5.083 | -5.445 |
| OR2F1 | -4.997 | -5.090 | -3.971 | -4.640 | -4.449 | -4.376 | -3.509 | -5.107 | -4.813 | -4.753 | -5.310 | -5.369 | -4.500 | -4.934 |
| OR2F2 | -5.121 | -4.665 | -3.793 | -4.117 | -4.049 | -4.057 | -3.265 | -4.818 | -4.256 | -4.200 | -5.046 | -4.735 | -4.049 | -4.505 |
| OR2G2 | -6.004 | -5.948 | -4.922 | -5.383 | -5.254 | -5.592 | -3.864 | -5.846 | -6.040 | -4.949 | -5.862 | -6.239 | -5.423 | -5.786 |
| OR2G3 | -5.402 | -4.993 | -4.371 | -4.586 | -4.561 | -4.726 | -3.637 | -5.208 | -4.816 | -4.710 | -5.502 | -5.423 | -4.772 | -5.048 |
| OR2G6 | -6.216 | -5.276 | -4.539 | -4.861 | -5.113 | -5.239 | -3.673 | -5.915 | -5.267 | -5.078 | -5.671 | -5.596 | -5.323 | -5.682 |
| OR2H1 | -5.296 | -5.335 | -4.473 | -4.757 | -5.207 | -5.220 | -3.696 | -5.366 | -5.318 | -5.301 | -5.732 | -5.545 | -5.262 | -5.662 |
| OR2H2 | -5.635 | -5.494 | -4.542 | -4.801 | -4.961 | -4.974 | -3.780 | -5.619 | -5.153 | -5.079 | -6.089 | -5.841 | -5.245 | -5.480 |
| OR2J2 | -5.418 | -4.834 | -4.310 | -4.587 | -4.743 | -4.850 | -3.409 | -5.495 | -4.770 | -4.749 | -5.339 | -5.256 | -5.044 | -5.071 |
| OR2J3 | -5.817 | -5.134 | -4.352 | -4.679 | -4.961 | -4.910 | -3.463 | -5.454 | -4.953 | -5.163 | -5.455 | -5.290 | -5.144 | -5.359 |
| OR2K2 | -4.950 | -4.916 | -3.910 | -4.335 | -4.616 | -4.094 | -3.598 | -4.337 | -4.239 | -4.702 | -5.075 | -5.050 | -4.359 | -4.650 |
| OR2L2 | -5.440 | -5.613 | -4.754 | -4.945 | -4.963 | -5.335 | -3.750 | -5.852 | -5.425 | -4.812 | -6.279 | -5.907 | -5.120 | -5.566 |
| OR2L3 | -5.721 | -5.334 | -4.371 | -4.761 | -4.957 | -5.051 | -3.722 | -5.562 | -5.104 | -4.830 | -5.804 | -5.641 | -5.026 | -5.307 |
| OR2L5 | -5.765 | -5.702 | -4.769 | -5.150 | -5.320 | -5.353 | -3.881 | -6.031 | -5.598 | -5.071 | -5.939 | -5.826 | -5.570 | -5.585 |
| OR2L8 | -6.014 | -5.540 | -4.782 | -5.060 | -5.313 | -5.432 | -3.736 | -5.831 | -5.516 | -5.195 | -6.012 | -5.882 | -5.495 | -5.784 |
| OR2LD | -5.487 | -5.464 | -4.478 | -4.807 | -4.817 | -5.287 | -3.437 | -5.877 | -5.342 | -4.984 | -5.895 | -5.818 | -5.334 | -5.638 |
| OR2M2 | -5.502 | -5.289 | -4.513 | -4.782 | -4.640 | -4.949 | -3.657 | -5.514 | -5.168 | -5.319 | -6.088 | -5.104 | -4.745 | -4.902 |
| OR2M3 | -5.157 | -5.064 | -4.172 | -4.523 | -4.752 | -4.681 | -3.216 | -5.053 | -4.861 | -4.810 | -5.328 | -5.191 | -4.769 | -5.017 |
| OR2M4 | -5.671 | -5.254 | -4.574 | -4.619 | -4.873 | -5.177 | -3.324 | -5.532 | -5.312 | -5.240 | -5.867 | -5.546 | -5.439 | -5.578 |
| OR2M5 | -5.098 | -4.684 | -3.553 | -4.015 | -4.066 | -3.945 | -3.034 | -4.791 | -4.291 | -4.178 | -4.864 | -5.039 | -4.365 | -4.353 |
| OR2M7 | -4.478 | -4.535 | -3.649 | -4.197 | -4.029 | -3.960 | -3.043 | -4.358 | -4.051 | -3.954 | -4.927 | -4.765 | -4.216 | -4.235 |
| OR2S1 | -5.393 | -5.126 | -4.266 | -4.710 | -4.713 | -4.615 | -3.313 | -5.111 | -4.878 | -4.930 | -5.684 | -5.391 | -4.763 | -4.833 |
| OR2T1 | -4.951 | -4.657 | -4.119 | -4.108 | -4.369 | -4.751 | -2.943 | -5.198 | -4.810 | -4.727 | -5.146 | -4.610 | -4.382 | -4.876 |
| OR2T2 | -4.887 | -4.499 | -3.757 | -3.992 | -4.040 | -4.194 | -3.034 | -4.433 | -4.319 | -4.243 | -4.856 | -4.769 | -4.108 | -4.581 |
| OR2T3 | -4.859 | -4.566 | -3.751 | -4.039 | -4.241 | -4.421 | -3.135 | -4.725 | -4.262 | -4.182 | -4.570 | -4.732 | -4.484 | -4.717 |
| OR2T4 | -5.315 | -5.108 | -4.327 | -4.339 | -4.522 | -4.668 | -3.665 | -5.181 | -4.877 | -4.645 | -5.458 | -5.421 | -4.905 | -4.968 |
| OR2T5 | -5.261 | -3.896 | -4.180 | -4.063 | -3.725 | -4.241 | -3.054 | -5.117 | -4.520 | -4.095 | -4.196 | -4.044 | -4.330 | -4.804 |
| OR2T6 | -4.741 | -4.417 | -4.087 | -4.116 | -3.976 | -4.052 | -3.309 | -4.764 | -4.539 | -4.178 | -4.743 | -4.627 | -4.504 | -4.597 |
| OR2T7 | -5.662 | -4.496 | -4.380 | -4.427 | -4.754 | -4.687 | -3.230 | -5.356 | -4.838 | -4.604 | -4.590 | -4.528 | -5.155 | -5.226 |
| OR2T8 | -5.108 | -4.709 | -4.035 | -4.404 | -4.505 | -4.633 | -3.204 | -5.054 | -4.837 | -4.465 | -5.245 | -4.997 | -4.634 | -4.941 |
| OR2V1 | -5.450 | -5.072 | -4.298 | -4.410 | -4.575 | -4.624 | -3.311 | -5.396 | -5.015 | -4.419 | -5.443 | -5.367 | -4.671 | -5.048 |
| OR2V2 | -5.245 | -4.743 | -4.170 | -4.240 | -4.354 | -4.448 | -3.386 | -5.033 | -4.627 | -4.433 | -5.266 | -5.135 | -4.507 | -4.733 |
| OR2W1 | -4.832 | -4.578 | -3.852 | -4.219 | -4.369 | -4.335 | -3.413 | -4.863 | -4.508 | -4.711 | -5.020 | -4.861 | -4.634 | -4.680 |
| OR2W3 | -4.929 | -4.586 | -3.741 | -4.065 | -4.263 | -4.182 | -3.242 | -4.446 | -4.377 | -4.417 | -4.929 | -4.861 | -4.178 | -4.431 |
| OR2W5 | -4.468 | -4.091 | -3.574 | -3.708 | -4.118 | -4.139 | -2.975 | -4.122 | -4.243 | -4.347 | -4.554 | -4.487 | -3.987 | -4.077 |
| OR2Y1 | -6.661 | -5.634 | -4.863 | -4.999 | -5.255 | -5.368 | -3.769 | -6.320 | -5.767 | -5.702 | -5.587 | -5.650 | -5.708 | -5.852 |
| OR2Z1 | -5.524 | -4.996 | -4.025 | -4.496 | -4.578 | -4.657 | -3.557 | -5.268 | -5.042 | -4.573 | -5.353 | -5.307 | -4.837 | -4.966 |
| OR3A1 | -5.481 | -4.796 | -4.271 | -4.343 | -4.503 | -4.793 | -3.411 | -5.431 | -5.181 | -4.780 | -5.190 | -5.146 | -5.192 | -4.992 |
| OR3A2 | -4.794 | -4.860 | -4.261 | -4.532 | -4.588 | -4.289 | -3.508 | -4.689 | -4.873 | -4.661 | -5.123 | -5.014 | -4.578 | -4.794 |
| OR3A3 | -5.478 | -4.609 | -3.894 | -4.313 | -4.227 | -4.428 | -3.304 | -5.223 | -4.741 | -4.484 | -5.063 | -4.889 | -4.591 | -4.737 |
| OR3A4 | -5.342 | -5.273 | -4.263 | -4.747 | -4.747 | -4.716 | -3.748 | -5.221 | -4.845 | -4.818 | -5.636 | -5.448 | -4.782 | -4.961 |
| OR4A5 | -5.403 | -4.704 | -4.279 | -4.575 | -4.580 | -4.808 | -3.677 | -5.122 | -4.991 | -4.863 | -4.648 | -4.968 | -4.972 | -4.969 |
| OR4B1 | -5.331 | -4.891 | -4.036 | -4.463 | -4.810 | -4.915 | -3.420 | -5.222 | -4.946 | -4.819 | -5.448 | -5.385 | -4.855 | -5.004 |
| OR4C3 | -4.528 | -4.859 | -4.246 | -4.623 | -4.587 | -4.770 | -3.646 | -4.880 | -4.989 | -4.712 | -4.153 | -5.013 | -4.850 | -4.472 |
| OR4C5 | -5.031 | -4.689 | -3.954 | -4.325 | -4.385 | -4.318 | -3.369 | -4.928 | -4.548 | -4.353 | -4.795 | -4.901 | -4.733 | -4.708 |
| OR4C6 | -5.043 | -4.951 | -4.019 | -4.210 | -4.191 | -4.275 | -3.170 | -4.830 | -4.712 | -4.450 | -5.373 | -5.139 | -4.553 | -4.648 |
| OR4CB | -5.322 | -5.231 | -4.137 | -4.625 | -4.577 | -4.736 | -3.616 | -5.552 | -4.817 | -4.778 | -5.301 | -5.349 | -4.970 | -4.913 |
| OR4CC | -3.771 | -5.305 | -4.261 | -4.841 | -4.751 | -4.692 | -3.740 | -4.171 | -4.569 | -4.591 | -4.910 | -5.303 | -4.855 | -5.180 |
| OR4CD | -5.080 | -4.635 | -4.247 | -4.772 | -4.324 | -4.483 | -3.563 | -4.938 | -4.574 | -3.997 | -4.746 | -4.900 | -4.537 | -4.829 |
| OR4CF | -5.649 | -4.982 | -4.297 | -4.411 | -4.706 | -4.891 | -3.438 | -5.129 | -5.241 | -4.971 | -5.405 | -5.309 | -4.968 | -5.236 |
| OR4CG | -5.124 | -5.113 | -3.965 | -4.693 | -4.443 | -4.506 | -3.676 | -5.118 | -4.946 | -4.527 | -5.415 | -5.399 | -4.516 | -4.749 |
| OR4D1 | -5.572 | -5.215 | -4.624 | -4.794 | -4.792 | -4.912 | -3.670 | -5.757 | -5.005 | -5.003 | -5.531 | -5.679 | -4.911 | -5.300 |
| OR4D2 | -5.011 | -5.123 | -4.427 | -5.003 | -4.640 | -4.443 | -3.947 | -5.090 | -4.734 | -4.672 | -5.503 | -5.453 | -4.810 | -5.020 |
| OR4D5 | -5.327 | -4.631 | -4.508 | -4.302 | -4.684 | -4.974 | -3.555 | -5.438 | -5.158 | -4.902 | -3.983 | -4.895 | -5.356 | -5.260 |
| OR4D6 | -5.278 | -4.781 | -4.069 | -4.422 | -4.173 | -4.493 | -3.587 | -5.078 | -4.463 | -4.452 | -5.324 | -5.063 | -4.464 | -4.628 |
| OR4D9 | -5.146 | -5.302 | -4.266 | -4.810 | -4.502 | -4.562 | -3.686 | -5.256 | -4.814 | -4.618 | -5.547 | -5.581 | -5.003 | -4.973 |
| OR4DA | -5.709 | -5.371 | -4.393 | -4.926 | -4.760 | -4.803 | -3.697 | -5.594 | -5.252 | -5.076 | -5.790 | -5.632 | -5.062 | -5.237 |
| OR4DB | -5.363 | -5.159 | -4.206 | -4.874 | -4.459 | -4.576 | -3.647 | -5.167 | -4.893 | -4.601 | -5.612 | -5.487 | -4.918 | -5.086 |
| OR4E2 | -5.407 | -4.787 | -4.282 | -4.374 | -4.552 | -4.638 | -3.356 | -5.474 | -5.031 | -4.841 | -4.897 | -5.089 | -4.875 | -4.859 |
| OR4F3 | -5.146 | -4.676 | -4.164 | -4.095 | -4.472 | -4.484 | -3.198 | -5.284 | -4.778 | -4.373 | -4.986 | -5.025 | -4.655 | -4.853 |
| OR4F4 | -4.744 | -4.500 | -3.968 | -4.125 | -4.113 | -4.343 | -3.226 | -4.521 | -4.492 | -4.187 | -4.564 | -4.456 | -4.544 | -4.750 |
| OR4F5 | -5.120 | -4.306 | -3.910 | -3.928 | -4.069 | -4.277 | -3.728 | -4.957 | -4.434 | -4.224 | -4.397 | -4.543 | -4.575 | -4.521 |
| OR4F6 | -5.934 | -4.692 | -4.576 | -4.687 | -4.887 | -4.803 | -3.785 | -5.508 | -5.015 | -4.647 | -4.007 | -4.771 | -4.847 | -5.094 |
| OR4K1 | -5.558 | -4.715 | -4.070 | -4.280 | -4.630 | -4.532 | -3.286 | -5.089 | -4.823 | -4.871 | -5.175 | -5.029 | -4.742 | -4.980 |
| OR4K2 | -4.613 | -3.801 | -4.011 | -4.123 | -4.393 | -4.601 | -3.444 | -4.530 | -4.680 | -4.506 | -3.234 | -3.935 | -4.634 | -4.810 |
| OR4K5 | 0.445 | -2.070 | -3.175 | -2.720 | -2.995 | -2.368 | -3.101 | -1.627 | -2.415 | -2.725 | -0.548 | -1.331 | -2.267 | -2.196 |
| OR4KD | -5.179 | -4.402 | -3.776 | -3.993 | -4.178 | -4.176 | -3.201 | -4.867 | -4.393 | -4.323 | -4.884 | -4.754 | -4.543 | -4.692 |
| OR4KE | -5.203 | -4.661 | -3.830 | -4.018 | -4.404 | -4.427 | -3.098 | -5.043 | -4.673 | -4.598 | -5.024 | -4.988 | -4.503 | -4.726 |
| OR4KF | -4.858 | -4.353 | -3.678 | -4.127 | -3.939 | -3.913 | -3.080 | -4.690 | -4.350 | -3.877 | -4.664 | -4.539 | -4.112 | -4.460 |
| OR4KH | -5.052 | -4.677 | -4.062 | -4.203 | -4.307 | -4.372 | -2.989 | -4.862 | -4.516 | -4.442 | -5.135 | -5.081 | -4.585 | -4.639 |
| OR4L1 | -5.008 | -4.452 | -3.773 | -3.967 | -4.344 | -4.287 | -3.117 | -4.765 | -4.440 | -4.380 | -4.856 | -4.747 | -4.077 | -4.588 |
| OR4M1 | -2.750 | -4.871 | -4.327 | -4.749 | -4.362 | -4.135 | -3.745 | -4.253 | -4.754 | -4.387 | -4.978 | -5.028 | -4.575 | -4.461 |
| OR4M2 | -5.012 | -4.511 | -3.984 | -4.654 | -4.485 | -4.624 | -3.752 | -4.927 | -4.570 | -4.569 | -4.307 | -4.753 | -4.857 | -4.850 |
| OR4N2 | -5.459 | -5.535 | -4.267 | -5.052 | -4.758 | -4.631 | -3.621 | -5.208 | -4.997 | -4.723 | -6.043 | -5.878 | -5.050 | -5.160 |
| OR4N4 | -5.158 | -4.934 | -4.183 | -4.410 | -4.252 | -4.687 | -3.496 | -5.031 | -4.777 | -4.489 | -5.316 | -5.148 | -4.441 | -4.836 |
| OR4N5 | -5.047 | -4.605 | -4.196 | -4.256 | -4.248 | -4.390 | -3.167 | -4.948 | -4.693 | -4.597 | -4.063 | -4.379 | -4.744 | -4.651 |
| OR4P4 | -5.282 | -5.149 | -4.512 | -4.676 | -4.963 | -5.065 | -3.707 | -5.396 | -5.090 | -4.424 | -5.093 | -5.102 | -5.344 | -5.174 |
| OR4Q3 | -5.080 | -4.973 | -4.288 | -4.461 | -4.728 | -4.696 | -3.425 | -5.145 | -4.942 | -4.482 | -4.779 | -5.190 | -5.260 | -5.062 |
| OR4S1 | -4.871 | -4.649 | -3.978 | -4.196 | -4.131 | -4.232 | -3.429 | -4.667 | -4.633 | -4.274 | -5.257 | -4.890 | -4.449 | -4.600 |
| OR4S2 | -3.443 | -5.380 | -4.423 | -4.894 | -5.018 | -4.750 | -3.937 | -4.256 | -4.693 | -4.193 | -4.798 | -5.129 | -4.872 | -4.779 |
| OR4X1 | -5.237 | -5.492 | -4.278 | -5.053 | -4.543 | -4.908 | -3.960 | -5.152 | -5.039 | -5.037 | -6.150 | -5.592 | -5.094 | -5.278 |
| OR4X2 | -5.288 | -4.590 | -4.522 | -4.125 | -4.752 | -4.712 | -3.123 | -4.899 | -4.896 | -4.676 | -5.036 | -4.862 | -4.514 | -4.768 |
| OR5A1 | -5.212 | -4.682 | -4.012 | -4.365 | -4.274 | -4.409 | -3.352 | -4.981 | -4.799 | -4.598 | -5.049 | -4.989 | -4.565 | -5.100 |
| OR5A2 | -5.372 | -5.067 | -4.082 | -4.574 | -4.500 | -4.579 | -3.367 | -5.200 | -4.689 | -4.676 | -5.575 | -5.353 | -4.621 | -4.880 |
| OR5B2 | -5.081 | -4.742 | -3.680 | -4.197 | -4.033 | -4.295 | -3.330 | -4.803 | -4.166 | -4.329 | -5.163 | -4.893 | -4.364 | -4.671 |
| OR5B3 | -5.511 | -4.789 | -4.365 | -4.380 | -4.592 | -4.785 | -3.418 | -5.694 | -5.011 | -4.683 | -5.290 | -5.159 | -4.858 | -5.197 |
| OR5BC | -5.192 | -4.532 | -3.806 | -4.315 | -4.439 | -4.600 | -3.463 | -5.103 | -4.522 | -4.568 | -4.796 | -4.757 | -4.645 | -4.767 |
| OR5BH | -5.005 | -4.963 | -4.131 | -4.852 | -4.595 | -4.689 | -3.739 | -5.250 | -4.806 | -4.491 | -5.176 | -5.125 | -4.537 | -4.936 |
| OR5C1 | -5.151 | -4.788 | -3.960 | -4.291 | -4.250 | -4.224 | -3.435 | -4.902 | -4.529 | -4.307 | -5.232 | -4.950 | -4.464 | -4.630 |
| OR5DD | -5.201 | -4.591 | -3.953 | -4.130 | -4.409 | -4.566 | -3.270 | -4.824 | -4.520 | -4.491 | -5.018 | -4.823 | -4.438 | -4.731 |
| OR5DE | -5.035 | -4.600 | -3.861 | -4.318 | -4.303 | -4.470 | -3.837 | -5.101 | -4.583 | -4.587 | -4.866 | -4.879 | -4.791 | -4.916 |
| OR5DG | -5.009 | -4.304 | -3.920 | -3.915 | -4.055 | -4.119 | -2.923 | -4.780 | -4.273 | -4.386 | -4.861 | -4.560 | -4.143 | -4.651 |
| OR5DI | -4.878 | -4.369 | -3.587 | -3.814 | -4.067 | -4.206 | -3.051 | -4.342 | -4.325 | -4.241 | -4.728 | -4.655 | -4.114 | -4.488 |
| OR5F1 | -5.604 | -5.434 | -4.440 | -4.664 | -4.948 | -5.021 | -3.645 | -5.645 | -5.177 | -4.876 | -5.508 | -5.362 | -5.095 | -5.196 |
| OR5H1 | -4.892 | -4.581 | -3.890 | -3.940 | -4.277 | -4.111 | -3.177 | -4.884 | -4.399 | -4.554 | -4.785 | -4.955 | -4.137 | -4.459 |
| OR5H2 | -5.205 | -4.701 | -4.077 | -4.183 | -4.324 | -4.545 | -3.195 | -5.098 | -4.721 | -4.505 | -4.922 | -4.854 | -4.359 | -4.842 |
| OR5H6 | -5.111 | -4.311 | -4.000 | -4.012 | -4.506 | -4.399 | -2.816 | -4.765 | -4.493 | -4.367 | -4.692 | -4.646 | -4.628 | -4.659 |
| OR5I1 | -5.244 | -4.588 | -4.013 | -4.241 | -4.281 | -4.393 | -3.199 | -4.976 | -4.685 | -4.356 | -5.054 | -5.049 | -4.777 | -4.719 |
| OR5J2 | -5.393 | -4.987 | -4.259 | -4.647 | -4.576 | -4.557 | -3.630 | -5.263 | -4.579 | -4.804 | -5.489 | -5.266 | -4.870 | -5.046 |
| OR5K1 | -5.132 | -4.709 | -3.997 | -4.583 | -4.408 | -4.625 | -3.711 | -5.102 | -4.491 | -4.523 | -5.200 | -4.945 | -4.530 | -4.864 |
| OR5K2 | -5.136 | -5.009 | -3.975 | -4.432 | -4.458 | -4.692 | -3.604 | -4.923 | -4.539 | -4.430 | -5.299 | -5.367 | -4.653 | -4.702 |
| OR5K3 | -4.008 | -5.270 | -4.392 | -5.085 | -5.044 | -5.125 | -3.932 | -4.965 | -4.947 | -4.734 | -3.711 | -4.826 | -5.355 | -5.200 |
| OR5K4 | -4.978 | -4.463 | -3.822 | -4.069 | -3.808 | -3.962 | -3.234 | -4.579 | -4.427 | -4.196 | -4.905 | -4.716 | -4.084 | -4.534 |
| OR5L1 | -5.585 | -5.074 | -4.627 | -4.575 | -4.979 | -5.067 | -3.341 | -5.571 | -5.227 | -5.228 | -5.348 | -5.542 | -5.299 | -5.564 |
| OR5L2 | -5.819 | -5.404 | -4.528 | -4.773 | -4.897 | -5.114 | -3.348 | -5.849 | -5.313 | -5.026 | -6.017 | -5.869 | -5.076 | -5.484 |
| OR5M1 | -5.255 | -4.722 | -4.089 | -4.189 | -4.178 | -4.393 | -3.085 | -5.060 | -4.641 | -4.446 | -5.183 | -5.017 | -4.807 | -4.757 |
| OR5M3 | -5.524 | -4.843 | -3.967 | -4.132 | -4.359 | -4.496 | -3.123 | -5.208 | -4.676 | -4.567 | -5.384 | -5.050 | -4.635 | -4.820 |
| OR5M8 | -5.082 | -4.585 | -4.068 | -4.325 | -4.315 | -4.410 | -3.179 | -4.922 | -4.878 | -4.412 | -5.120 | -5.145 | -4.812 | -4.771 |
| OR5M9 | -5.142 | -4.476 | -3.840 | -4.083 | -4.324 | -4.502 | -2.986 | -5.033 | -4.362 | -4.350 | -4.825 | -4.822 | -4.395 | -4.682 |
| OR5MA | -5.195 | -4.578 | -4.104 | -4.117 | -4.285 | -4.231 | -3.021 | -4.708 | -4.615 | -4.417 | -5.123 | -4.879 | -4.789 | -4.595 |
| OR5MB | -5.059 | -4.410 | -3.935 | -4.207 | -4.315 | -4.433 | -3.338 | -4.981 | -4.596 | -4.349 | -5.157 | -4.718 | -4.717 | -4.644 |
| OR5P2 | -5.452 | -4.449 | -3.944 | -4.235 | -4.305 | -4.377 | -3.156 | -5.050 | -4.751 | -4.497 | -5.004 | -4.721 | -4.812 | -4.661 |
| OR5P3 | -5.562 | -5.367 | -4.397 | -4.789 | -4.835 | -5.103 | -3.831 | -5.574 | -5.034 | -5.055 | -6.075 | -5.656 | -4.936 | -5.474 |
| OR5T1 | -5.071 | -4.994 | -3.834 | -4.534 | -4.142 | -4.092 | -3.613 | -4.604 | -4.418 | -4.154 | -5.333 | -5.143 | -4.570 | -4.448 |
| OR5T2 | -4.973 | -4.892 | -3.953 | -4.611 | -4.314 | -4.266 | -3.552 | -4.936 | -4.292 | -4.334 | -5.385 | -5.145 | -4.714 | -4.651 |
| OR5T3 | -5.686 | -5.554 | -4.640 | -5.025 | -5.049 | -5.375 | -3.699 | -6.052 | -5.375 | -5.277 | -5.836 | -5.928 | -5.418 | -5.416 |
| OR5V1 | -5.144 | -5.359 | -4.123 | -4.675 | -4.733 | -4.823 | -3.472 | -4.751 | -4.897 | -4.824 | -5.781 | -5.477 | -4.924 | -4.831 |
| OR5W2 | -4.647 | -4.565 | -4.228 | -4.209 | -4.552 | -4.440 | -3.067 | -4.694 | -4.662 | -4.314 | -4.830 | -4.585 | -4.765 | -4.816 |
| OR6A2 | -5.438 | -4.888 | -4.301 | -4.598 | -4.440 | -4.715 | -3.456 | -5.239 | -4.957 | -4.826 | -5.504 | -5.280 | -4.669 | -5.153 |
| OR6B1 | -4.108 | -4.061 | -3.610 | -3.712 | -3.577 | -3.674 | -2.969 | -3.969 | -4.064 | -3.748 | -4.379 | -4.199 | -3.920 | -4.028 |
| OR6B2 | -5.135 | -4.820 | -4.193 | -4.226 | -4.549 | -4.465 | -3.094 | -5.139 | -4.640 | -4.534 | -5.318 | -5.081 | -4.665 | -5.275 |
| OR6B3 | -4.411 | -4.502 | -3.943 | -4.014 | -4.313 | -4.108 | -3.196 | -4.428 | -4.026 | -3.845 | -4.334 | -4.539 | -3.976 | -4.095 |
| OR6C1 | -5.347 | -4.664 | -4.122 | -4.086 | -4.600 | -4.581 | -2.961 | -5.006 | -4.774 | -4.893 | -5.140 | -4.724 | -4.714 | -5.222 |
| OR6C2 | -4.691 | -4.286 | -3.753 | -3.832 | -3.980 | -4.210 | -3.091 | -4.563 | -4.155 | -4.340 | -4.474 | -4.479 | -4.292 | -4.729 |
| OR6C3 | -5.219 | -4.707 | -4.233 | -4.142 | -4.907 | -4.875 | -3.127 | -5.015 | -4.813 | -4.859 | -5.015 | -4.865 | -4.983 | -5.123 |
| OR6C4 | -5.371 | -5.236 | -4.173 | -4.806 | -4.579 | -4.771 | -3.420 | -4.805 | -4.766 | -4.884 | -5.808 | -5.592 | -4.962 | -4.878 |
| OR6C6 | -5.336 | -5.145 | -4.197 | -4.496 | -4.641 | -4.484 | -3.345 | -4.896 | -4.882 | -4.595 | -5.429 | -5.417 | -4.793 | -4.862 |
| OR6F1 | -4.920 | -4.634 | -4.073 | -4.377 | -4.275 | -4.448 | -3.245 | -4.339 | -4.327 | -4.245 | -4.946 | -4.846 | -4.325 | -4.689 |
| OR6J1 | -6.006 | -5.262 | -4.525 | -4.916 | -5.026 | -5.227 | -3.652 | -6.048 | -5.331 | -5.494 | -5.657 | -5.555 | -5.448 | -5.575 |
| OR6K2 | -5.554 | -5.487 | -4.439 | -4.807 | -5.010 | -5.140 | -3.518 | -5.310 | -5.138 | -5.120 | -5.407 | -5.598 | -5.256 | -5.266 |
| OR6K3 | -5.477 | -4.883 | -4.411 | -4.401 | -4.786 | -4.931 | -3.584 | -5.237 | -4.989 | -4.933 | -5.305 | -5.075 | -5.150 | -5.069 |
| OR6K6 | -5.084 | -4.609 | -4.078 | -4.151 | -4.224 | -4.467 | -3.470 | -4.990 | -4.482 | -4.432 | -4.742 | -5.045 | -4.418 | -4.645 |
| OR6M1 | -6.074 | -5.509 | -4.728 | -4.995 | -5.451 | -5.348 | -3.727 | -5.983 | -5.298 | -5.490 | -5.688 | -5.806 | -5.434 | -5.618 |
| OR6N1 | -5.099 | -5.897 | -4.648 | -5.187 | -5.260 | -5.323 | -3.773 | -5.340 | -5.520 | -5.257 | -6.206 | -5.698 | -5.615 | -5.544 |
| OR6N2 | -5.863 | -5.656 | -4.823 | -5.187 | -5.270 | -5.166 | -3.843 | -5.880 | -5.412 | -5.131 | -5.659 | -5.895 | -5.813 | -5.303 |
| OR6P1 | -5.318 | -5.159 | -4.363 | -4.898 | -4.842 | -4.895 | -3.457 | -5.208 | -4.966 | -4.880 | -5.899 | -5.544 | -4.846 | -4.982 |
| OR6Q1 | -5.045 | -4.550 | -3.815 | -4.121 | -4.165 | -3.966 | -3.045 | -4.447 | -4.293 | -4.166 | -4.899 | -4.800 | -4.204 | -4.462 |
| OR6S1 | -5.912 | -5.332 | -4.645 | -4.748 | -5.152 | -5.160 | -3.289 | -5.936 | -5.175 | -5.215 | -5.685 | -5.698 | -5.349 | -5.560 |
| OR6T1 | -6.115 | -4.998 | -4.484 | -4.622 | -5.088 | -5.277 | -3.457 | -5.718 | -5.194 | -5.378 | -5.303 | -5.503 | -5.227 | -5.455 |
| OR6V1 | -5.916 | -5.301 | -4.323 | -4.642 | -4.812 | -5.028 | -3.494 | -5.760 | -5.379 | -5.070 | -5.531 | -5.685 | -5.097 | -5.221 |
| OR6X1 | -5.431 | -5.008 | -4.588 | -4.527 | -5.047 | -4.845 | -3.399 | -5.211 | -4.819 | -4.977 | -5.059 | -5.000 | -4.735 | -5.130 |
| OR6Y1 | -4.979 | -4.625 | -3.917 | -4.034 | -4.640 | -4.879 | -3.271 | -5.141 | -4.508 | -4.378 | -4.677 | -5.027 | -4.572 | -4.848 |
| OR7A5 | -5.099 | -4.974 | -3.793 | -4.329 | -4.339 | -4.488 | -3.356 | -5.062 | -4.547 | -4.621 | -5.239 | -5.118 | -4.231 | -4.727 |
| OR7AA | -4.595 | -4.538 | -3.871 | -4.090 | -4.111 | -4.087 | -3.328 | -4.237 | -4.391 | -4.206 | -4.710 | -4.811 | -4.406 | -4.459 |
| OR7AH | -4.671 | -4.574 | -3.697 | -4.024 | -4.152 | -4.167 | -3.126 | -4.283 | -4.116 | -4.176 | -4.987 | -4.771 | -4.152 | -4.387 |
| OR7C1 | -5.282 | -4.252 | -4.258 | -3.935 | -4.609 | -4.654 | -3.181 | -5.044 | -4.889 | -4.611 | -4.819 | -4.535 | -4.686 | -4.682 |
| OR7C2 | -4.798 | -4.302 | -3.696 | -3.814 | -4.115 | -4.171 | -3.043 | -4.830 | -4.350 | -4.436 | -4.765 | -4.626 | -4.333 | -4.430 |
| OR7D2 | -5.064 | -4.588 | -3.940 | -3.992 | -4.215 | -4.312 | -3.181 | -4.517 | -4.393 | -4.364 | -4.875 | -4.761 | -4.340 | -4.540 |
| OR7D4 | -4.958 | -4.300 | -3.672 | -3.884 | -4.033 | -4.197 | -2.857 | -4.802 | -4.401 | -4.035 | -4.630 | -4.585 | -4.652 | -4.434 |
| OR7G1 | -4.695 | -4.636 | -3.679 | -4.294 | -3.916 | -3.775 | -3.282 | -4.668 | -4.024 | -4.201 | -4.999 | -4.801 | -3.984 | -4.112 |
| OR7G2 | -4.612 | -4.370 | -3.907 | -3.822 | -4.093 | -3.979 | -3.074 | -4.469 | -4.461 | -4.090 | -4.550 | -4.471 | -4.261 | -4.314 |
| OR7G3 | -5.184 | -4.499 | -3.806 | -4.098 | -4.312 | -4.467 | -2.949 | -4.695 | -4.511 | -4.524 | -4.793 | -4.589 | -4.714 | -4.651 |
| OR8A1 | -4.601 | -4.523 | -3.845 | -4.065 | -4.289 | -4.209 | -3.106 | -4.595 | -4.276 | -4.114 | -5.027 | -4.842 | -4.509 | -4.490 |
| OR8B2 | -5.521 | -5.038 | -3.978 | -4.366 | -4.349 | -4.640 | -3.070 | -5.019 | -4.697 | -4.514 | -5.356 | -5.392 | -4.534 | -4.867 |
| OR8B3 | -5.280 | -4.690 | -4.026 | -4.283 | -4.230 | -4.603 | -3.254 | -5.058 | -4.738 | -4.489 | -5.241 | -4.928 | -4.540 | -4.813 |
| OR8B4 | -5.341 | -4.730 | -4.279 | -4.262 | -4.567 | -4.659 | -3.171 | -5.087 | -4.768 | -4.651 | -5.200 | -5.012 | -4.587 | -4.862 |
| OR8B8 | -5.924 | -5.302 | -4.596 | -4.680 | -5.023 | -4.971 | -3.247 | -5.842 | -5.309 | -5.148 | -5.692 | -5.623 | -5.283 | -5.459 |
| OR8BC | -5.380 | -4.364 | -4.197 | -4.199 | -4.579 | -4.573 | -3.216 | -5.231 | -4.625 | -4.569 | -4.966 | -4.687 | -4.780 | -4.958 |
| OR8D1 | -5.663 | -5.081 | -4.323 | -4.657 | -4.812 | -4.872 | -3.640 | -5.938 | -5.111 | -5.045 | -5.424 | -5.296 | -5.331 | -5.372 |
| OR8D2 | -5.963 | -5.547 | -4.283 | -4.757 | -4.707 | -4.918 | -3.771 | -5.698 | -5.005 | -4.938 | -5.819 | -5.458 | -5.050 | -5.426 |
| OR8D4 | -5.065 | -4.407 | -3.764 | -3.875 | -4.232 | -4.350 | -2.901 | -4.795 | -4.483 | -4.229 | -4.929 | -4.745 | -4.337 | -4.626 |
| OR8G1 | -4.970 | -4.585 | -3.738 | -4.117 | -4.259 | -4.333 | -2.963 | -4.638 | -4.621 | -4.259 | -5.093 | -4.724 | -4.554 | -4.730 |
| OR8G5 | -4.965 | -4.642 | -3.629 | -4.134 | -4.164 | -4.063 | -3.297 | -4.944 | -4.451 | -4.157 | -5.047 | -5.031 | -4.247 | -4.429 |
| OR8H1 | -4.975 | -4.890 | -3.979 | -4.408 | -4.397 | -4.370 | -3.376 | -5.048 | -4.725 | -4.573 | -5.348 | -5.220 | -4.689 | -4.979 |
| OR8H2 | -5.346 | -5.016 | -4.198 | -4.597 | -4.689 | -4.768 | -3.607 | -5.237 | -4.884 | -4.851 | -5.604 | -5.420 | -4.910 | -5.006 |
| OR8H3 | -4.995 | -4.916 | -3.971 | -4.507 | -4.319 | -4.294 | -3.390 | -4.755 | -4.604 | -4.251 | -5.340 | -5.285 | -4.653 | -4.778 |
| OR8I2 | -5.187 | -4.598 | -3.876 | -4.118 | -4.343 | -4.590 | -3.023 | -5.004 | -4.421 | -4.520 | -5.055 | -4.898 | -4.517 | -4.905 |
| OR8J1 | -5.036 | -4.783 | -3.898 | -4.438 | -4.136 | -4.540 | -3.230 | -4.869 | -4.589 | -4.571 | -5.365 | -5.051 | -4.681 | -4.871 |
| OR8J3 | -4.585 | -4.572 | -3.676 | -3.973 | -4.168 | -4.190 | -3.117 | -4.517 | -4.337 | -4.223 | -4.907 | -5.029 | -4.627 | -4.888 |
| OR8K1 | -4.801 | -4.682 | -4.019 | -4.267 | -4.373 | -4.563 | -3.312 | -5.063 | -4.592 | -4.360 | -5.269 | -4.953 | -4.648 | -4.894 |
| OR8K3 | -5.396 | -4.863 | -4.309 | -4.412 | -4.512 | -4.574 | -3.418 | -4.768 | -4.958 | -4.372 | -5.167 | -5.059 | -4.668 | -4.972 |
| OR8K5 | -5.079 | -4.684 | -3.937 | -4.337 | -4.158 | -4.276 | -3.388 | -4.699 | -4.508 | -4.272 | -5.301 | -5.018 | -4.386 | -4.751 |
| OR8S1 | -4.963 | -4.440 | -3.960 | -4.123 | -4.282 | -4.338 | -3.235 | -4.809 | -4.551 | -4.520 | -4.803 | -4.742 | -4.576 | -4.744 |
| OR8U1 | -5.422 | -4.695 | -3.967 | -4.096 | -4.472 | -4.443 | -3.376 | -5.042 | -4.559 | -4.566 | -5.061 | -4.936 | -4.774 | -5.250 |
| OR8U3 | -5.170 | -4.382 | -3.807 | -3.955 | -4.094 | -4.345 | -3.291 | -4.818 | -4.302 | -4.339 | -4.805 | -4.662 | -4.488 | -4.573 |
| OR8U8 | -5.278 | -4.805 | -3.893 | -4.147 | -4.426 | -4.617 | -3.201 | -4.934 | -4.618 | -4.477 | -4.692 | -4.825 | -4.605 | -5.027 |
| OR8U9 | -4.631 | -4.430 | -3.757 | -4.156 | -4.029 | -4.020 | -3.046 | -4.398 | -4.129 | -4.055 | -4.491 | -4.377 | -4.260 | -4.292 |
| OR9A2 | -4.839 | -4.512 | -3.933 | -4.013 | -4.062 | -4.205 | -3.123 | -4.645 | -4.382 | -4.226 | -4.777 | -4.804 | -4.506 | -4.619 |
| OR9A4 | -5.890 | -5.435 | -4.689 | -4.861 | -5.137 | -5.400 | -3.742 | -5.996 | -5.487 | -4.995 | -5.169 | -5.737 | -5.417 | -5.508 |
| OR9G1 | -6.036 | -5.382 | -4.314 | -4.713 | -4.978 | -4.907 | -3.683 | -5.643 | -5.255 | -5.173 | -5.829 | -5.659 | -4.655 | -5.444 |
| OR9G4 | -5.566 | -4.514 | -3.908 | -4.262 | -4.267 | -4.502 | -3.388 | -5.232 | -4.614 | -4.513 | -4.996 | -4.809 | -4.588 | -4.791 |
| OR9G9 | -5.014 | -5.016 | -4.122 | -4.558 | -4.467 | -4.419 | -3.488 | -4.971 | -4.705 | -4.470 | -5.163 | -5.306 | -4.723 | -4.794 |
| OR9I1 | -5.096 | -4.664 | -3.681 | -4.329 | -4.303 | -4.068 | -3.139 | -4.584 | -4.711 | -4.385 | -5.009 | -4.982 | -4.586 | -4.785 |
| OR9K2 | -5.592 | -4.761 | -4.138 | -4.202 | -4.608 | -4.721 | -3.046 | -5.333 | -4.803 | -4.714 | -5.246 | -4.994 | -4.859 | -4.877 |
| OR9Q1 | -4.926 | -4.640 | -4.401 | -4.774 | -4.569 | -4.433 | -3.866 | -4.544 | -4.550 | -4.511 | -4.800 | -4.898 | -4.858 | -4.924 |
| OR9Q2 | -5.152 | -4.669 | -3.964 | -4.274 | -4.449 | -4.491 | -3.180 | -5.172 | -4.622 | -4.545 | -5.093 | -4.962 | -4.565 | -4.611 |
